# IgE and T Cell Reactivity to a Comprehensive Panel of Cockroach Allergens in Relation to Disease

**DOI:** 10.3389/fimmu.2020.621700

**Published:** 2021-02-10

**Authors:** Anna Pomés, Véronique Schulten, Jill Glesner, Ricardo da Silva Antunes, Aaron Sutherland, Leonard B. Bacharier, Avraham Beigelman, Paula Busse, April Frazier, Alessandro Sette

**Affiliations:** ^1^ Basic Research, Indoor Biotechnologies, Inc., Charlottesville, VA, United States; ^2^ Center for Infectious Disease and Vaccine Research, La Jolla Institute for Immunology, La Jolla, CA, United States; ^3^ Department of Pediatrics, Washington University School of Medicine, St. Louis, MO, United States; ^4^ Department of Pediatrics, Vanderbilt University Medical Center, Nashville, TN, United States; ^5^ Kipper Institute of Allergy and Immunology, Schneider Children’s Medical Center of Israel, Tel Aviv University, Tel Aviv, Israel; ^6^ Division of Clinical Immunology, Icahn School of Medicine at Mount Sinai, New York, NY, United States; ^7^ Department of Medicine, University of California San Diego, La Jolla, CA, United States

**Keywords:** cockroach allergy, IgE, T-cell reactivity, rhinitis, asthma, diagnosis, immunotherapy

## Abstract

IgE sensitization to cockroach allergens is associated with development of allergic diseases, such as asthma. To understand the relevance of different cockroach allergens for diagnosis and immunotherapy, a comprehensive analysis of IgE antibody levels and T cell reactivity to an expanded set of cockroach allergens and their relationship to disease was performed in a cohort of USA cockroach sensitized patients. IgE antibody levels to recombinant chitinase and hemocyanin were measured for 23 subjects by custom-made ImmunoCAPs and compared with IgE levels to eight cockroach allergens we previously reported for the same cohort. *Ex vivo* T cell activation (Ox40/PDL-1 expression) of PBMCs stimulated with peptide pools derived from 11 German cockroach proteins, including nine official cockroach allergens, plus chitinase and vitellogenin, was determined by flow cytometry. IgE prevalences to chitinase (17%) and hemocyanin (44%) were comparable to values for the other eight allergens that we previously reported (21–57%). Hemocyanin (Bla g 3), was a major allergen (one to which more than 50% of patients with an allergy to its source react) for a sub-group of 15 highly cockroach-sensitized subjects (IgE > 3.5 kU_A_/L: 53%). Chitinase was officially named as new allergen Bla g 12. Cockroach-specific IgE levels in plasma showed excellent correlation with the sum of 10 allergen-specific IgE (r = 0.94, p < 0.001). T cell reactivity to 11 proteins was highly variable among subjects, the highest being for vitellogenin, followed by Bla g 3. The main finding was that cockroach allergen-specific IgE and T cell reactivity patterns were unique per subject, and lacked immunodominant allergens and correlation with clinical phenotype/disease severity in the studied cohort. Knowing the subject-specific B/T cell reactivity profiles to a comprehensive panel of cockroach allergens will contribute to diagnosis of cockroach allergy and will be important for planning and assessing allergen immunotherapy outcomes, according to the allergen content in therapeutic cockroach extracts.

## Introduction

Cockroach allergy is an important health problem associated with the development of asthma, especially in inner-cities where cockroach infestations frequently occur (1, 2). In the USA and temperate areas of the world, the German cockroach *Blattella germanica* is the most common species associated with allergy. Exposure by inhalation to allergens released by the cockroach into the environment can lead to IgE production (sensitization) in susceptible individuals. However, currently immunotherapy is not used for cockroach allergy and there are no standardized extracts for therapy. To date, no strong immunodominant allergen/s regarding IgE prevalence have been described for German cockroach in a given population (3, 4), in contrast with other allergen sources such as cat (Fel d 1) or house dust mite (Der p 1 and Der p 2). There is evidence of differences in allergen immunodominance for IgE antibody and T cell reactivity for few allergens (5). For example, while Bla g 2 and Bla g 5 are dominant allergens in terms of IgE responses in USA patients (3), Bla g 2 represents a minor target for allergic T cells (5, 6). Conversely, some antigens which are dominantly recognized by T cells (e.g., NBGA5) were reported to be minor targets for IgE responses from cockroach-sensitized donors (6). In addition, each cockroach-sensitized patient has an individual profile of B and T cell reactivity to different molecules in cockroach extracts (3, 4, 7). This observation is of relevance, since it is currently unknown whether the immunotherapeutic potency of cockroach extracts resides in the modulation of T cells, antibody/B cells, or both. It is possible that by selecting cockroach extracts for immunotherapy based on IgE reactivity alone, extracts that are poor stimulators for T cells are selected, potentially leading to variability and inconsistencies in clinical trial results. In addition, different patterns of T and B cell reactivity might be associated with different clinical outcomes. Indeed, previous data from cockroach-sensitized donors showed that T cells from rhinitis-only sensitized donors recognized a different set of allergens and epitopes, as compared to the T cells derived from asthmatic patients (6). In another study, focusing on B cell reactivity, we showed that in a cohort of 10 year-old children allergen-specific IgE levels were higher and recognized a broader range of allergens among cockroach allergic subjects with asthma and rhinitis than among cockroach allergic subjects without those diseases (8).

In 2005, Satinover et al. showed that the IgE reactivity to five cockroach allergens (from groups 1, 2, 4, 5, and 7) was variable among cockroach allergic patients (n = 118), with unique subject sensitization profiles (3). Bla g 2 and Bla g 5, the most prevalent allergens (54 and 37%, respectively), were not recognized by IgE from a large number of individuals tested. Interestingly, 36% of cockroach allergic subjects did not recognize any of the five allergens, which suggested that other, not yet identified allergens, might be involved in cockroach IgE sensitization. To address this possibility, we analyzed an expanded set of eight German cockroach allergens that incorporated groups 6, 9, and 11 in two recent studies (4, 8). In the first one, a highly significant correlation between cockroach specific IgE levels and the sum of all eight allergen-specific IgE levels was found, but 17% (4/23) of the patients did not react to any of the allergens (4). A second study showed once more that few subjects did not react to the eight allergens tested (23%: 11/47) (8). These observations prompted us to investigate the allergenicity of three additional cockroach proteins for which some evidence of B and/or T cell reactivity had been reported among cockroach-sensitized subjects: hemocyanin, chitinase and vitellogenin. The B and T cell reactivity to these proteins was analyzed in comparison to allergens previously identified (from groups 1, 2, 4, 5, 6, 7, 9, and 11) and listed in the official database maintained by the World Health Organization/International Union of Immunological Societies (WHO/IUIS) Allergen Nomenclature Sub-Committee (www.allergen.org).

Hemocyanin from insect hemolymph was first described as major allergen in the American cockroach *Periplaneta americana* in Taiwan, resulting in four polymorphisms that are currently listed as Per a 3 in the above mentioned official Allergen Nomenclature database (9, 10). A homolog from German cockroach, Bla g 3, was also reported in the USA, but as minor allergen with an IgE prevalence of 22% (11).

Chitinase is an enzyme in the gastrointestinal tract of cockroaches. It is essential for digestion of chitin by hydrolyzing the N-acetyl-D-glucosamine 1,4-β-linkages of chitin polymers (12). Chitinase purified from the midgut of American cockroaches was reported to be a 45 kDa allergen recognized by serum IgE from 63.8% (30/47) of cockroach allergic patients using immunoblots and skin prick test. This allergen was named Per a 12 (12). Chitinase allergens were also found in house dust mite allergens, and named Der p 15 and Der f 15 (13, 14). Here, Bla g 3 and a chitinase from *Blattella germanica* listed in GenBank were expressed, purified, and analyzed for IgE antibody binding in comparison with eight previously reported cockroach allergens.

Vitellogenin was identified as a German cockroach allergen in Taiwan in 46.88% (15/32) of cockroach allergic subjects (15). Insect vitellogenins are large proteins (~200 kDa) synthesized in the fat body. *In vivo*, they are secreted into the hemolymph and after receptor-mediated endocytosis by oocytes they are stored as vitellins or yolk proteins, as reserve food-source for the future embryo (16). Other functions have been found for vitellogenin in honeybee workers, that usually do not lay eggs, related to food signaling, food-related behavior, immunity, stress resistance and longevity (17). Api m 12 and Ves v 6 are vitellogenin allergens (18).

In addition to IgE antibody analysis, the current study measured the T cell reactivity to an expanded set of 11 cockroach proteins (including 10 allergens plus vitellogenin), aiming to compare and identify the most relevant molecules in terms of B and T cell reactivity, and their association to disease. The ultimate goal was to gain insight into immunological responses associated with cockroach allergy for improving diagnosis and treatment, especially in light of the current cockroach immunotherapy trial from the Inner City Asthma Consortium (CRITICAL).

## Materials and Methods

### Subjects

A cohort of 23 subjects sensitized to cockroach (IgE titer ≥ 0.35 kU_A_/L) were recruited from San Diego, California; St Louis, Missouri; and New York, New York, according to institutional review board approval (protocols VD-112-0217, 201305110, and GCO 13-0691) (**Table 1**). This is the same cohort that was analyzed in our previous study (4). All had a history of allergy symptoms to cockroach, and most had asthma, rhinitis, or both. Eligibility criteria and disease severity assessment were based on participant-reported history of asthma, allergic rhinitis, or both for at least one year prior to recruitment using a questionnaire. All subjects enrolled in this study provided written consent. IgE antibody titers were determined from plasma by using the ImmunoCAP system (Thermo Fisher Scientific, Uppsala, Sweden). Seventy percent of subjects were female, mean age was 39 ± 10 years, and cockroach-specific IgE titers were 16.46 ± 22.76 kU_A_/L (range, 0.91-76.20 kU_A_/L) on average. Two negative controls were a non-allergic individual and a subject sensitized to Fel d 1, Bet v 1, and Phl p 5, but not to cockroach (IgE < 0.1 kU_A_/L using i6 ImmunoCAP). Both were negative for Bla g 3 and Bla g 12.

**Table 1:** Information of cockroach allergic donors.

Subject #	Donor ID	CR-specific IgE (kU_A_/L)	Total T cell response	SPT Wheal size (mm)	Clinical severity	Age	Gender
23	1445	76.2	3,276	9	AR/Severe Asthma	32	F
22	1277	66.2	327	10	AR/Intermittent Asthma	53	M
21	1228	56.5	7,803	7	AR/Moderate Asthma	54	F
20	1424	45.2	896	10	AR/Moderate Asthma	30	F
19	1425	36	n.d	7	AR/Severe Asthma	39	F
18	1446	17.3	305	7.5	AR (no asthma)	50	M
17	1229	12.2	3,943	9	AR/Moderate Asthma	49	M
16	1398	10.5	n.d.	8.5	AR/Moderate Asthma	30	F
15	2210	10.13	2,227	n.d.	AR (Asthma Status Unknown)	28	F
14	1437	8.32	n.d.	9	Mild Asthma	38	F
13	1175	7.27	987	3	AR (no asthma)	43	F
12	1406	5.3	149	4.5	AR/Intermittent Asthma	41	F
11	1864	4.82	322	n.d.	AR (Asthma Status Unknown)	37	F
10	1257	4.78	206	6	AR/Severe Asthma	37	F
9	1231	4.47	180	3.5	AR/Severe Asthma	23	F
8	2083	3.41	4,789	n.d.	AR (Asthma Status Unknown)	23	F
7	1665	2.24	3,487	n.d.	AR (Asthma Status Unknown)	26	F
6	1365	2.01	1,538	4.5	AR/Intermittent Asthma	49	F
5	1006	1.32	8,954	0	Moderate/Severe Asthma	44	M
4	1367	1.27	560	6	AR (no asthma)	37	F
3	2196	1.23	652	n.d.	Very mild AR (Asthma Status Unknown)	53	M
2	1439	0.94	483	8	Moderate/Severe Asthma	32	M
1	1441	0.91	545	0	Mild Asthma	47	M
	Average	16.46	2,081.4			38.9	69.6% F
	Std deviation	22.76	2,580.9			9.8	30.4% M

Cockroach-specific IgE, sum of allergen-specific T cell responses (Tact/ 10^6^ CD4+), skin prick test wheal size, clinical severity, age, and sex of the study cohort of 23 subjects sensitized to cockroach. Darker color means more disease severity (red: allergic rhinitis and asthma, blue: allergic rhinitis, green: asthma only).

AR, Allergic rhinitis; n.d., not determined.

### Expression and Purification of Cockroach Allergens

Three German cockroach proteins were expressed in *Pichia pastoris* using the pPICZαB vector, by methanol induction. The proteins were chitinase, hemocyanin (Bla g 3.0101) and an N-terminal fragment of vitellogenin named N-vitellogenin (18-322 with 6-His-tag). The rational for expression of the N-terminal vitellogenin is explained below. Their expected MW calculated from the amino acid sequence including the C-terminal 6-His-tag were 58.1 kDa for chitinase (without the 23 amino acid signal peptide), 79.6 kDa for hemocyanin and 36.3 kDa for the N-terminal vitellogenin (GenBank accession numbers KJ789158, GU086323, and CAA06379, respectively) (11, 19, 20).

Chitinase and Bla g 3 were purified by metal affinity chromatography (20 mM phosphate 0.5 M NaCl 20 mM imidazole pH 7.4, 20 mM phosphate 0.5 M NaCl 500 mM Imidazole pH 7.4). N-vitellogenin was purified by metal affinity chromatography followed by size exclusion chromatography (20 mM TrisBase 0.02 M NaCl pH 7.2). A purity of >90% was confirmed for the three molecules on silver-stained SDS-PAGE (data not shown). Purity of chitinase and N-vitellogenin was additionally confirmed by mass spectrometry (98 and 93%, respectively). Mass spectrometry was performed with a Thermofisher Q Exactive mass spectrometer, equipped with a Vanquish Flex UHPLC liquid chromatography system. Samples were digested with trypsin and reduced with tris(2-carboxyethyl) phospine (TCEP), followed by alkylation with iodoacetamide. The LC-MS/MS analysis was performed using ThermoFisher Proteome Discoverer 2.2 software, employing the Sequest HT search engine.

### Rationale for Expression of an N-Terminal Vitellogenin Fragment

IgE antibody binding to the full vitellogenin could not be tested because attempts to express the full-length protein from German cockroach (MW I18-N1862) resulted in a fragmented recombinant protein. Proteolytic cleavage of vitellogenin also occurs *in vivo*, typically close to polyserine sequences at an RXXR consensus sequence motif by subtilisin-like endoproteases (21, 22), leading to the formation of different size units, depending on the insect group. In lamprey, for example, the resulting chains remain associated after cleavage to form the lipid-binding lipovitellin-phosvitin complex, which is a source of amino acids, lipids, phosphate, and cations during embryogenesis (23, 24). Based on this information about fragmentation of vitellogenin *in vivo*, an N-terminal part of vitellogenin (the N-sheet domain) from German cockroach was expressed.

The rational for the expression of an N-terminal fragment of vitellogenin (N-vitellogenin) was that: a) an equivalent 40 kDa unit had been isolated from the abdominal fat body tissue of honeybees (17), b) a reported structural model showed that this 40 kDa unit folded into a defined β-sheet (17), and c) the structure in which the model was based, the lamprey lipovitellin-phosvitin complex, showed that the N-sheet (Q17-V296) had limited contact with the rest of vitellogenin (**Supplemental Figure 1**) (23, 24). Therefore, these three observations suggested that independent expression of this correctly folded unit might be feasible. The N-sheet domain of the German cockroach vitellogenin was expressed in *Pichia pastoris*, comprising the amino acids I18 to S322 plus a C-terminal 6xHis-tag for purification by nickel affinity chromatography. The recombinant N-vitellogenin was >90% pure in silver-stained SDS-PAGE gel and further shown to be 93% pure by mass spectrometry. Residues 1–17 were not included since they are the putative signal peptide according to the SignalP-5.0 server (http://www.cbs.dtu.dk/services/SignalP-5.0/) and Comas et al. (20). The expected MW of the 311 amino acid construct with the His-tag was 36,300.02 da.

### Biotinylation and Optimization of Biotinylation of Cockroach Allergens

EZ-Link Sulfo-NHS-LC-Biotin (Thermo Scientific, Rockford, IL) was added to a defined amount of each allergen (2 mg for chitinase, 0.25 mg for Bla g 3) at a 10-fold molar excess and incubated for 30 min at room temperature. The biotinylated mix was put over a pre-washed Zeba Desalt Spin Column (Thermo Scientific, Rockford, IL) two times and the concentration was determined after biotinylation by Advanced Protein Assay (Cytoskeleton, Denver, Colorado).

The quantification of biotinylation was carried out by using a Quant Tag™ Biotin Kit (Vector Laboratories, Burlingame, CA). Samples were tested in triplicate against a known biotin standard curve to determine the number of biotins per allergen molecule.

### Optimization of Biotinylated Allergen Loaded to the Streptavidin ImmunoCAP

Streptavidin ImmunoCAPs (Thermo Fisher Scientific, Portage, MI) were loaded and incubated on a Phadia 100, with the biotinylated allergen at amounts ranging from 0.5 to 10 µg/CAP. Two different human plasma samples from individuals allergic to the allergen (that had been originally tested for IgE binding to 2–3 µg/CAP) were selected for optimization experiments. Their IgE levels to the allergen-loaded CAPs were measured in a Phadia 250 following manufacturer’s instructions (Thermo Fisher Scientific, Portage, MI) to select optimal amount of biotinylated allergen to be loaded to the streptavidin ImmunoCAPs.

### Measurement of Allergen-Specific IgE Antibody Levels to Bla g 3 and Bla g 12

Biotinylated cockroach allergens Bla g 3 and Bla g 12 were loaded and incubated on streptavidin ImmunoCAPs using the Phadia 100. The ImmunoCAPs were transferred to the Phadia 250, where measurements of allergen-specific IgE antibody binding were performed according to manufacturer’s instructions.

### IgE Antibody Binding to N-Terminal Vitellogenin

IgE antibody binding N-vitellogenin was assessed by chimeric enzyme-linked immunosorbent assay. Microtiter plates were coated at 4°C overnight with 10 μg/ml of N-vitellogenin. Plate was blocked for 30 min with 1% bovine serum albumin/phosphate-buffered saline, 0.05% Tween 20, pH 7.4. A 1 h incubation with sera (dilutions 1:2 and 1:10) was performed. Bound IgE was detected using biotin-labeled goat anti-human IgE (Kirkegaard and Perry Laboratories, Gaithersburg, MD) at a 1:4,000 dilution (1h incubation), and quantified using a human/mouse chimeric IgE antibody as standard (25). Streptavidin peroxidase (1:1000) was added, followed by development using 2,2’-azino-bis(3-ethylbenzothiazoline-6-sulphonic acid) (ABTS) in 70 mM citrate phosphate buffer, pH 4.2 and 1:1,000 dilution of H_2_O_2_. Absorbance was read at 405 nm on a Bio-Tek EL800 Microplate Reader (Bio-Tek Instruments, Inc., Winooski, VT).

IgE binding to N-vitellogenin was tested in 22 of the cohort patients, and two additional plasma from: 1) a patient sensitized to dust mite but not to cat, dog, and cockroach that was used as an additional negative control, and 2) a patient sensitized to Fel d 1, Can f 1, Bet v 1 and Phl p 5, with cockroach-specific IgE of 2.34 kU_A_/L, had IgE against N-vitellogenin and served as positive control for the immunoassay. These two plasma were obtained from PlasmaLab International (Everett, WA, USA), which operates in full compliance of US Food and Drug Administration. Informed donor’s consent was obtained from each subject before the first donation. None of the patients tested (n = 22) from the cohort had IgE specific for N-vitellogenin.

### PBMC Isolation

Peripheral blood mononuclear cells (PBMCs) were isolated from whole blood by density gradient centrifugation according to the manufacturer’s instructions (Ficoll-Paque Plus, Amersham Biosciences, Uppsala, Sweden) as previously described (26). Cells were suspended in fetal bovine serum (FBS) containing 10% (vol/vol) dimethyl sulfoxide (DMSO) and cryopreserved in liquid nitrogen until further use.

### Peptide Synthesis

Sequences of 11 cockroach proteins (mostly known allergens) were collected from UniProt. A strategy using 15-mer peptides overlapping by 10 amino acids was selected and peptide sequences generated to get the full coverage of the antigen (**Supplemental Table 1**). A strategy to generate and test peptides with a larger overlap was considered but deemed not feasible in terms of peptides and number of existing cells available to perform the tests. Peptides were purchased from A&A (San Diego, CA, USA) as crude material on a small (1 mg) scale. Individual peptides were resuspended in DMSO at a final concentration of 40 mg/ml. The peptides were pooled, lyophilized, and the resulting pool of peptides for each allergen was resuspended to a final concentration of 1 mg/ml/peptide.

### Activation Induced Marker (AIM) Assay

*Ex vivo* T cell responses were measured based on T cell activation assays previously described (27, 28). This assay detects cells that are activated as a result of antigen specific stimulation by upregulation of activation-induced surface markers. Here, we assessed dual expression of OX40 (CD134) and PDL-1. Briefly, PBMC were thawed and rested overnight, plated at 1×10^6^ cells per well in a round-bottom 96-well plate. The next morning, cells were stimulated with peptide pools (2 μg/ml/peptide) for each individual allergen, PHA and PT (positive controls), or DMSO (negative control). Cells were incubated for 24 h. After the incubation, cells were labeled with a cocktail of antibodies (**Supplemental Table 2**). After staining and washing, flow cytometry was performed. Cells were acquired using a BD LSR II flow cytometer and data were analyzed using FlowJo software (TreeStar, Ashland, OR, USA). Geometric mean values with geometric SDs are shown for each of the individual allergens.

## Results

### IgE Reactivity to Chitinase and Hemocyanin From German Cockroach

**Figure 1:**
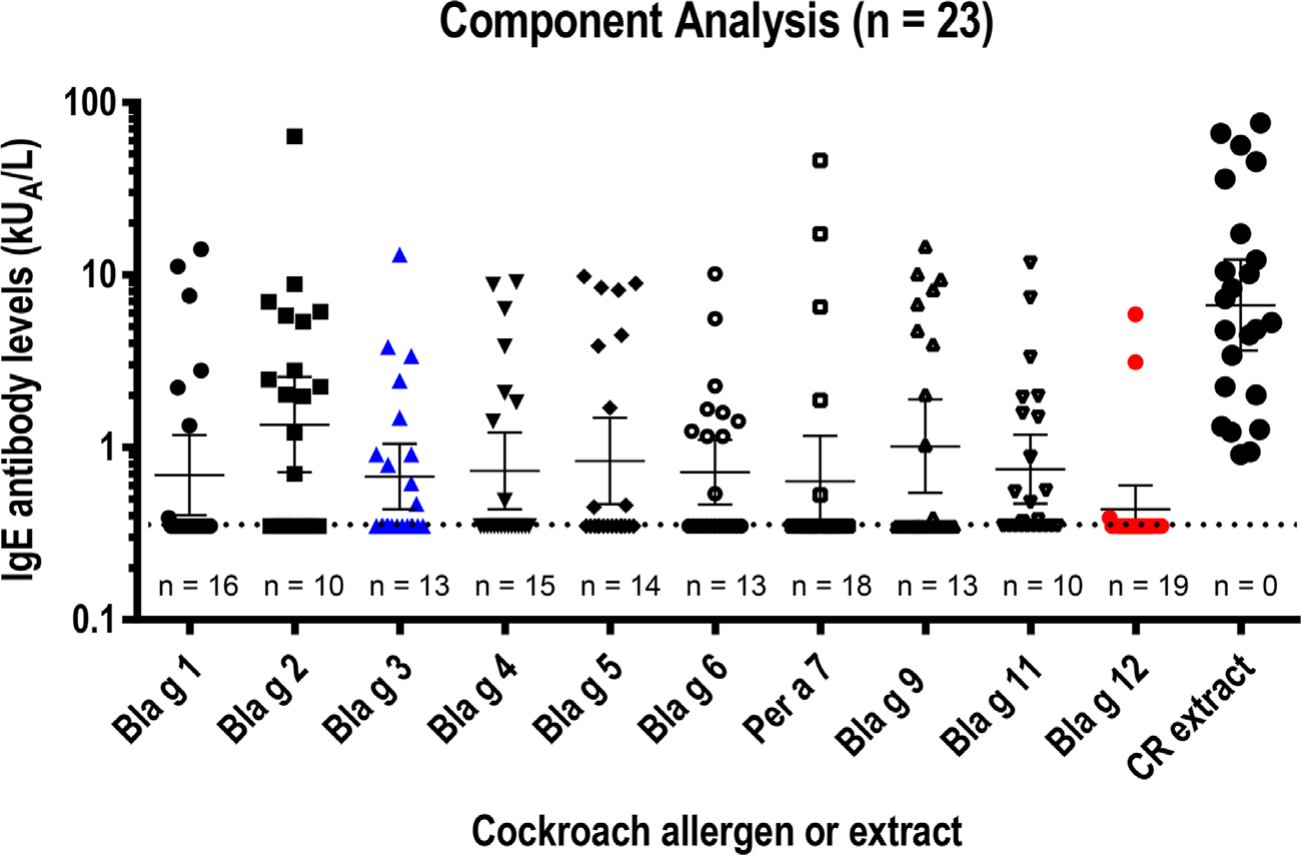
IgE reactivity to 10 cockroach allergens and cockroach extract in a USA cohort (n = 23). Long and short horizontal lines indicate geometric means and 95% CIs, respectively. The cut-off level for IgE quantification (0.35 kU_A_/L) is indicated by the horizontal dotted line. The number of negative results (< 0.35 kU_A_/L) is provided for each allergen under the corresponding cluster of symbols. Data obtained with Bla g 3 (blue) and Bla g 12 (red) -measured in an equivalent manner to the values previously reported for the other allergens and the same cohort (4)- were inserted in a plot showing IgE to the other eight allergens (black). IgE levels to the 10 allergens were combined in one plot to facilitate their visual comparison, because IgE levels are consistent for the same sera (properly stored at -20˚C) when measured in different occasions by ImmunoCAP, which has a high allergen capacity (several micrograms per CAP).

To assess the relevance of hemocyanin (Bla g 3) and chitinase, the IgE antibody levels to both purified molecules were measured and compared with the IgE reactivities to 8 other cockroach allergens that we previously reported for the same cohort (n = 23) (4) (**Figure 1**). A purity of >95% for both allergens was estimated on silver-stained SDS-PAGE (data not shown), and a 98% purity was additionally confirmed for chitinase by mass spectrometry. Details of the cohort, including cockroach-specific IgE, skin prick test wheal size and T cell reactivity, are shown in **Table 1**. The geometric mean of Bla g 3-specific IgEs (0.68 kU_A_/L) was similar to the average of geometric means of the 10 cockroach allergen-specific IgEs (0.78 kU_A_/L), and approximately half the maximum value, which was for Bla g 2 (1.36 kU_A_/L) (**Figure 1**). The lowest geometric mean of allergen-specific IgE was for Bla g 12 (0.44 kU_A_/L). There was a highly significant correlation between cockroach-specific IgE and the sum of 10 allergen-specific IgE levels (r = 0.94, p < 0.001; for log10 transformed data: r = 0.87, P < 0.001; n = 23). With individual sensitization profiles, there was overall a correlation of the number of allergens recognized per subject and their titer with the IgE antibody binding to the cockroach extract.

The prevalences of IgE sensitization among the 23 subjects tested were 43.5% (10/23) for Bla g 3 and 17.4% (4/23) for chitinase, using a conservative cut-off of 0.35 kU_A_/L (**Table 2**). The equivalent values using a lower cut-off (0.1 kU_A_/L) were 47.8% (11/23) and 30.4% (7/23), respectively. Based on these data, the chitinase was proven to be an allergen and was submitted to the WHO/IUIS Allergen Nomenclature database, which approved the assignment of this new allergen to group 12. Chitinase is now listed as Bla g 12 in the official database, and this name will be used from now on in this article.

**Table 2:** Frequencies of subjects with IgE and T cell reactivity.

		Frequencies of IgE and T cell responses to 10 allergens										
		Bla g 1	Bla g 2	Bla g 3	Bla g 4	Bla g 5	Bla g 6	Per a 7	Bla g 9	Bla g 11	Bla g 12	Average± Stdev
**IgE ≥ 0.35 kU/L** **(n = 23)**	**Number of subjects**	7	13	10	8	9	10	5	10	13	4	8.9 ± 3.0
	**Percentage of subjects (%)**	30.4	56.5	43.5	34.8	39.1	43.5	21.7	43.5	56.5	17.4	38.7 ± 13.0
**IgE ≥ 3.5 kU/L** **(n = 15)**	**Number of subjects**	7	11	8	7	7	9	5	8	11	3	7.6 ± 2.5
	**Percentage of subjects (%)**	46.7	73.3	53.3	46.7	46.7	60.0	33.3	53.3	73.3	20.0	50.7 ± 16.4
**T cell reactivity**	**Frequency of response (%)**	40	25	29	15	20	15	15	40	35	25	25.9 ± 9.9

Frequencies of subjects with IgE antibody specific for Bla g 3 and Bla g 12, compared to the ones previously reported for eight cockroach allergens (marked in grey). Frequencies of subjects with T cell responses to the 10 allergens tested.

The IgE prevalence of Bla g 3 (43.5%) was above the average of IgE prevalences for the 10 allergens tested (38.7 ± 13.0%), and for Bla g 1, Bla g 4, Bla g 5, and Per a 7 (Per a 7.0102, highly cross-reactive and sharing 98.6% amino acid identity with Bla g 7.0101) (n = 23) (**Table 2**). It was also under the corresponding values for Bla g 2 and Bla g 11 (57%) and the same as the ones for Bla g 6 and Bla g 9, previously reported for the same cohort (4). For a sub-group of highly allergic subjects with IgE ≥ 3.5 kU_A_/L (CAP class 3; n = 15) the IgE prevalences were 53.3 and 20.0%, respectively, regardless of cut-off. In subjects with cockroach-specific IgE > 5 kU_A_/L, the IgE prevalences were 58.3% for Bla g 3 and 16.7% for Bla g 12 (0.35 kU_A_/L cut-off), and 66.7% and 33.3% (0.1 kU_A_/L cut-off), respectively. These results indicate that Bla g 3 was a major allergen for this sub-group of highly cockroach-allergic subjects, adding to the other four major allergens (Bla g 2, Bla g 6, Bla g 9, and Bla g 11) (**Table 2**).

### Patterns of IgE Antibody Levels to 10 Cockroach Allergens

**Figure 2:**
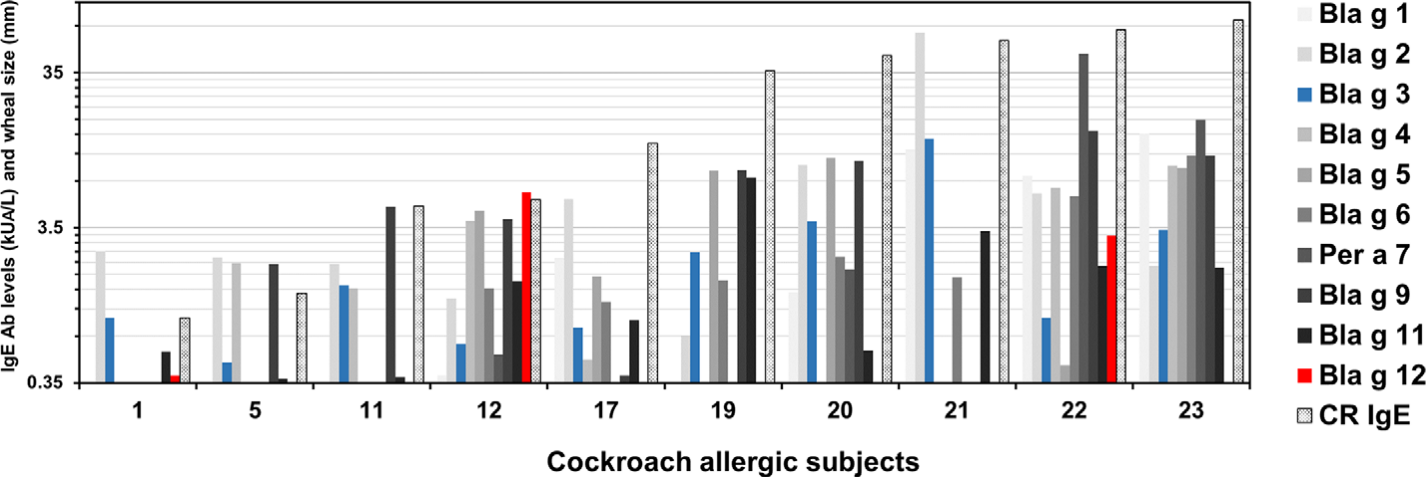
Patterns of IgE sensitization to 10 cockroach allergens. Patterns are shown only for 10 (out of the 23 tested) subjects that recognized Bla g 3. Subjects were numbered from 1 (1441) to 23 (1445) (from lowest to highest cockroach-specific IgE levels) as shown in **Table 1**. IgE data for Bla g 3 (blue) and Bla g 12 (red) are shown next to data previously measured in an equivalent manner and reported for the other eight allergens (different tones of grey) for comparison (4).

Patterns of IgE reactivity to the 10 cockroach allergens were variable in the cohort. Among 10 (out of 23) subjects that recognized Bla g 3, four (1, 11, 12, and 22) also recognized Bla g 12 (**Figure 2**). None of the allergens was dominant in this population. At the individual level, immunodominance was observed for one or few different allergens (**Table 3**). The number of subjects for which the highest IgE levels were for Bla g 2 was six, followed by four and three subjects for which the highest responses were for Bla g 9 and Bla g 5, respectively. There was only one subject with dominant IgE levels to either Bla g 4, Bla g 6, Bla g 11, or Bla g 12, whereas four subjects did not react to any of the 10 allergens (**Table 3**).

**Table 3:** Number of subjects with highest IgE and T cell responses.

	Bla g 1	Bla g 2	Bla g 3	Bla g 4	Bla g 5	Bla g 6	Per a 7	Bla g 9	Bla g 11	Bla g 12	Vitello genin	None	Total
**IgE to**	0	6	0	1	3	1	2	4	1	1	n.d.	4	23
**T cell response to**	0	0	3	0	0	1	4	1	0	0	11	0	20

Values indicate the number of subjects for which the highest IgE or T cell responses was for the allergens indicated in the top row.

### T Cell Reactivity to a Panel of 11 Allergens From German Cockroach

**Figure 3:**
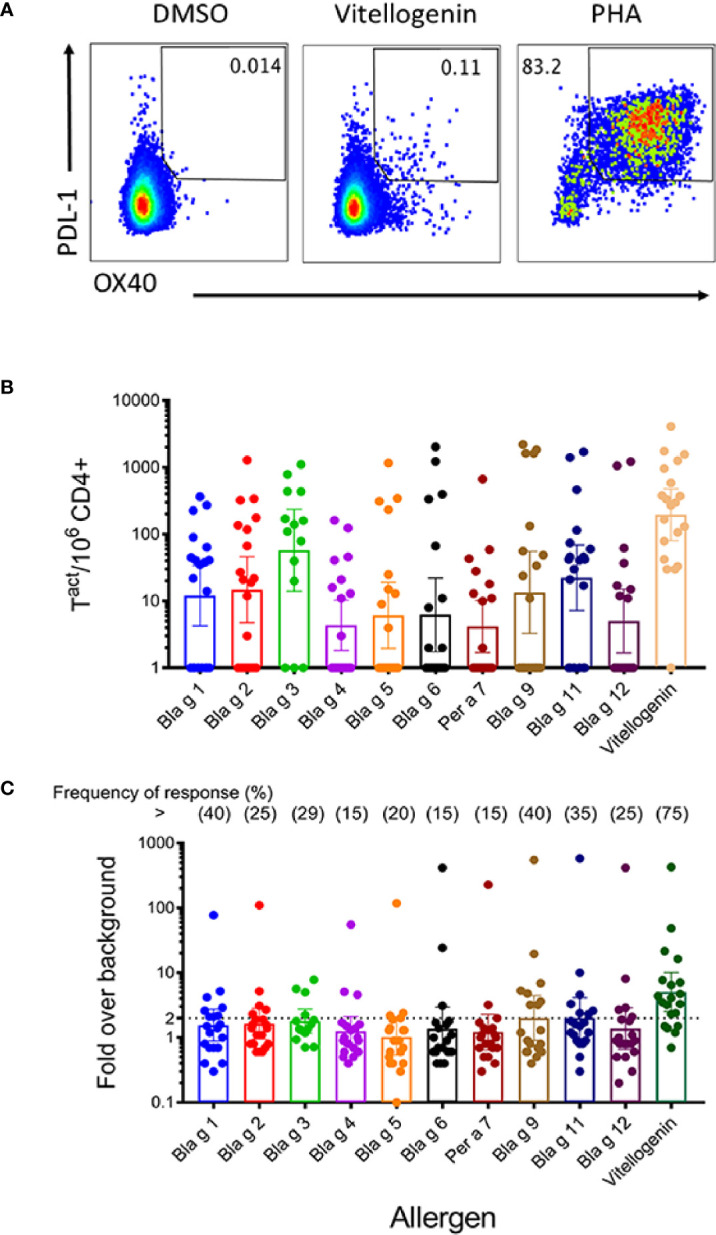
T cell activation in response to short-term allergen peptide pool stimulation. PBMC were stimulated with cockroach allergen-derived peptide pools for 24 h. Subsequently, T cell activation (Ox40/PDL-1 expression) was measured by flow cytometry. **(A)** Representative FACS plots showing Ox40/PDL-1 expression in response to medium, vitellogenin peptide pool and PHA stimulation. **(B, C)**: Graph bars showing allergen pool-specific T cell reactivity (absolute numbers) **(B)** and T cell responses expressed as fold increase over medium **(C)**. Frequency of response for each allergen is depicted on top. Each dot represents a donor. Geometric means with 95% CI are shown (n = 14 for Bla g 3, and n = 20 for the other 10 allergens).

The overall goal of this analysis was to examine whether there were CD4+ T cell responses specific for a set of different cockroach allergens (from groups 1, 2, 3, 4, 5, 6, 7, 9, 11, 12 and vitellogenin). The evaluations were based on previously described Activation Induced Marker (AIM) assays (27, 28), utilizing the OX40 and PDL-1 markers. A full list of antibodies used in these experiments is shown in **Supplemental Table 2**. To be able to separately establish the allergen specificity of the T cell responses, individual allergen sets of overlapping peptides were tested. Each set contained peptides spanning the whole sequence of each unique cockroach allergen (**Supplemental Table 1**). The allergens were selected on the basis of previous studies that characterized the responses of human subjects to cockroach allergens (4, 6, 7). Thus, in total 11 different peptide pools were tested in each donor, each corresponding to a different cockroach allergen (**Figure 3**). **Figure 3A** shows data from a representative donor, depicting the detection of OX40+PDL-1+ producing cells for vitellogenin. We also utilized a previously described MP as additional control encompassing T cell epitopes derived from ubiquitous *Bordetella pertussis* (PT) vaccine antigens. Good signal was observed in response to the positive control PHA (and PT, see also **Supplemental Figure 2**) but not detected in response to the negative (DMSO) control or in the case of donors unresponsive for a particular allergen. An overview of T cell reactivity across all donors is shown in the graphs where each donor is represented by a dot, and each bar represents the geometric mean response for each individual allergen expressed either as the absolute number per million of CD4+ T cells (**Figure 3B**) or as the fold change over the negative control (**Figure 3C**). The overall magnitude of T responses varied significantly across different subjects in the study cohort (ranging from 149 to 8,954 cells/million of CD4+ T cells; **Table 1**) consistent with what has been reported before for allergic responses against CR allergens (6, 7). In terms of the individual allergen response, vitellogenin reactivity was associated with the highest magnitude among all the allergens tested as well as the highest frequency of response (75% of the donors). The highest response observed for vitellogenin in terms of absolute numbers was followed by Bla g 3, Bla g 11, Bla g 1, and Bla g 2 (285, 125, 42, 29, and 20 activated cells per million CD4+ cells, respectively). In term of stimulation index (SI) represented as fold over background, the highest T cell response was also vitellogenin (5.06-fold activated cells per million CD4+ cells) and followed by Bla g 9 and Bla g 11 (both 2.01), and Bla g 3 (1.8). The lowest value was marginal and for Bla g 5 (1.02) (**Supplemental Table 3**).

### Lack of Association Between B and T Cell Reactivity, and Clinical Phenotype/Severity

No correlation was observed between the prevalences of IgE sensitization and the frequencies of the T cell response to 10 cockroach allergens at the population level in the cohort tested (**Table 2**), or between allergen-specific IgE antibody levels and magnitude of the T cell response at individual level (**Table 3**; **Supplemental Figure 3**). In fact, the subjects with the highest allergen-specific T cell responses were different than the ones with the highest IgE antibody levels: 11 subjects had the highest T cell responses to vitellogenin, followed by four and three subjects with the highest responses to Per a 7 and Bla g 3, respectively, one to Bla g 6 and one to Bla g 9 (**Table 3**). These results highlight the unique profiles of IgE and T cell responses to this large set of cockroach allergens.

The clinical phenotypes of the subjects in the cohort ranged from mild asthma or allergic rhinitis alone, to more severe forms of these diseases, or their combination (**Table 1**). Interestingly, there was no correlation between clinical phenotypes/severity and cockroach-specific IgE levels or T cell reactivity. For example, subjects with high level of disease severity (e.g., allergic rhinitis and moderate to severe asthma), had different cockroach-specific IgE levels, not necessarily always high (76.2, 36, 4.78, 4.47 kU_A_/L). Moderate to severe asthma alone was found in individuals with low cockroach-specific IgE (1.32, 0.94 kU_A_/L). Conversely, mild asthma was found associated to relatively high cockroach-specific IgE (8.32 kU_A_/L), in addition to low levels (0.91 kU_A_/L).

No allergen was specifically associated with severity of the disease. For example, four patients with the most severe disease (allergic rhinitis and severe asthma) had the highest levels of IgE to an allergen from either group 2, 7, 9, or none of the 10 tested. The allergen to which more subjects had the highest IgE levels was Bla g 2 (six subjects). Among those, there was not a clear association between Bla g 2-specific IgE levels and severity of the disease: whereas three out of those six with the highest levels of IgE to Bla g 2 (63.6, 6.1 and 5.36 kU_A_/L) had allergic rhinitis and moderate to severe asthma, among the three subjects with the lowest IgE levels (2.25 to 2.79 kU_A_/L), two had mild asthma and one moderate to severe asthma.

## Discussion

The goal was to expand our knowledge about the relative importance of cockroach allergens at the IgE and T cell level in relation to disease (asthma, rhinitis) for diagnosis and therapy. This is especially relevant for component analyses of clinical immunotherapy trials, given the variability of allergen content in extracts used for treatment, which may impact clinical efficacy (4, 29). The current study compares B (IgE) and T cell reactivity to an expanded set of proteins from cockroach. Cockroach allergy is associated with IgE sensitization to multiple allergens, none of which have been found to be consistently immunodominant in studied cockroach allergic populations. At the B cell level, the highest prevalence of IgE sensitization among cockroach allergic subjects was originally described for Bla g 2 (54–71%) (3, 30). This is in contrast with the existence of highly prevalent allergens from other sources, such as Fel d 1 from cat or Der p 1 and Der p 2 from house dust mite, with >90% IgE sensitization rates. In recent years, proteomic studies identified new IgE antibody binding proteins in cockroach, but their relevance for sensitization to the German cockroach in the USA is unknown (15, 31). At the T cell level, variable reactivity to several cockroach allergens has been reported, and new cockroach proteins not previously known as allergens (e.g. NBGA5), were found to induce T cell responses (6, 7). These observations prompted us to investigate additional cockroach proteins, such as hemocyanin, chitinase and vitellogenin, for which some evidence of B and/or T cell reactivity had been reported among cockroach sensitized subjects (6, 11, 12). IgE reactivity to chitinase and hemocyanin was compared with the corresponding values for 8 cockroach allergens that we previously reported in the same cohort of cockroach allergic subjects (4, 8). The capacity of the 10 allergens to induce activation of T cell responses was assessed by measuring the upregulation of two well defined co-stimulatory molecules (OX40 and PDL-1) in response to allergen-specific peptide pools. In addition, T cell reactivity was also measured to vitellogenin, because a closely homologous protein called NBGA5 has previously been reported to induce high T cell responses (6).

The low frequency of allergen specific T cell responses usually requires *in vitro* expansion steps to allow their characterization on a global scale. Although this approach allows for greater sensitivity, it may alter the phenotype of responding T cells. We and others previously demonstrated that allergen-specific T cells can be detectable *ex vivo* using a novel assay strategy with the combination of pools of T cell epitopes. This technique uses the upregulation of the activation markers as a read-out for T cell reactivity (AIM assay) (27), and can be further combined with intracellular cytokine staining (ICS) to further identify T cell phenotypes (32), but a larger number of cells is necessary. We thus decided to perform the AIM assay without ICS, due to our limitation in cell numbers and the fact that previous allergic studies have found that responses captured by this T-cell readout are majorly Th2 responses (33–35). This approach, commonly known as AIM, allows to identify antigen specific cells with high-specificity and high-sensitivity, and in an agnostically fashion (i.e., irrespective of their cytokine profile).

Although an extensive analysis of the tertiary and quaternary structures of the allergens was not performed, there was evidence that the allergens were properly folded for IgE measurements. First, the allergens tested had the expected size in native and SDS-PAGE gels (data not shown), suggesting a correct folding of the molecules. Second, the pPICZαB vector used for protein expression in *Pichia pastoris* has the secretion signal sequence from the *Saccharomyces cerevisiae* α-factor prepro peptide (36, 37). *P. pastoris* has been reported as a successful expression system for optimal expression of heterologous proteins, with the benefit of appropriate protein folding (38). In our experience, unlike *E. coli* expressed proteins that often require refolding, most proteins expressed in *P. pastoris* using the α-factor secretion signal are correctly folded and secreted to the media. Third, in particular for Bla g 3, this is a homologous protein to arthropod hemocyanins, which are hexameric oxygen-carrying proteins. The degree of oligomerization of the expressed Bla g 3 is unknown. Nevertheless, it is not expected to affect antibody binding for the following reasons. Several IgE antibody binding epitopes have been reported for the homolog allergen Per a 3, which are located on the surface of the hexamer (39). A surface location of IgE epitopes would also be expected for Bla g 3 since subjects would have been sensitized to the oligomer to which they were presumably exposed. In this case, the IgE antibodies binding to the hexamer should also bind to the monomer (if no major conformational differences affecting IgE epitopes exist between monomers and oligomers). Finally, the sum of IgE to 10 allergens correlates with IgE to cockroach extract that contain the natural allergens. The fact that subjects’ IgE recognized the molecules tested, and conformational epitopes are important for inhalant allergens, point to the correct folding of the proteins expressed.

The main observation was that all the cockroach proteins tested induced B and T cell reactivity. This result supports a link between IgE and T cell reactivity in German cockroach allergy, as was previously reported for fewer allergens (6). However, at a population level, no immunodominant allergens were identified for B or T cell reactivity in the analyzed cohort. There was no correlation between the prevalences of IgE sensitization and the frequencies of the T cell response to the 10 allergens, both of which are indicators of immunodominance in the allergic population. These results are in agreement with previous studies in cockroach and other allergies (5, 6, 40). Bla g 2, with the same low T cell response (25%) as chitinase, was a major allergen in this USA cohort regarding IgE reactivity (57–73%). The highest prevalence of IgE recognition (63%) was also found for Bla g 2 in Taiwan, but not in other parts of the world (15). In Brazil, 42% (24/57) of cockroach allergic patients had positive skin prick test to Per a 7, whereas the IgE reactivity to Per a 1, Per a 7, Bla g 2, Bla g 4, and Bla g 5 was remarkably low (≤ 7%) (41, 42). This observation could reflect cross-reactivity with either mite tropomyosin (80% identical to the cockroach homolog), and/or tropomyosin from intestinal parasites, particularly *Ascaris lumbricoides*, as it also occurs in Africa (41, 43). Levels of allergen exposure are major determinants of IgE sensitization (44). Cockroach allergen concentrations differ depending on the environment, as reported for 10 components in Taiwan (45). Therefore, differences in allergen-specific IgE sensitization are expected to occur in different environments according to the subject’s allergen exposure level.

At the individual level, a unique profile of IgE sensitization and T cell responses to 10 allergens showed dominance of different allergens per subject. The four patients with IgE specific for chitinase and hemocyanin (#1, 11, 12, and 22) were not the ones with the highest cockroach-specific IgE. For subject #12, chitinase dominated the response. However, overall, there was a correlation of the number of allergens recognized and their titer with the IgE antibody binding to the cockroach extract, as reported previously for eight allergens (4). Unlike other allergen sources, cockroach does not produce a dominant allergen. Subjects are sensitized to several allergens, and all the ones tested (not only one or few dominant ones) seem to contribute in different degrees to the IgE to cockroach extract. This leads to the excellent correlation observed between the sum of allergen-specific IgE and the cockroach-specific IgE. No correlation was observed between the levels of allergen-specific IgE and the magnitude of the allergen-specific T cell responses. For example, despite similarly low IgE prevalence (17.4%) and frequency of T cell responses to chitinase (only 25% of the subjects), the individual with the highest IgE level to chitinase (5.92 kU_A_/L; subject #12) did not correspond to the subject with the highest T cell response (fold activated T cells) to this protein (#14). Conversely, the patients with the highest T cell reactivity to Bla g 3 (subject #13) or Bla g 12 (#5), did not have IgE against these two allergens. Similarly, the subject with the highest T cell response to Bla g 2 (#5) did not have the highest Bla g 2-specific IgE (#18). These results indicate the existence of different individual allergen immunodominance for either B or T cell reactivity. They also indicate that a variability in subjects’ reactivity to extracts used for immunotherapy is expected, according to their allergen composition.

Vitellogenin was the protein that activated T cells the most. It is also the largest of the proteins tested, therefore the pool with the largest number of peptides. It is a possibility that larger antigens can be more immunogenic simply because they contain more epitopes. Therefore, the reactivity to a higher number of peptides spanning the entire sequence of a large antigen/allergen could elicit a higher magnitude of T cell responses. However, this is not always the case. For example, in the set of allergens described in our manuscript and for this particular cohort, the T cell reactivity for Bla g 11 is higher than the T cell reactivity for Chitinase (Bla g 12) despite similar number of peptides per pool (101 and 102 peptides, respectively). Current evidence also shows that allergen T cell dominance varies in other cohorts, as reported for Bla g 5, Bla g 9 and Bla g 11 (6) (and data not shown). Insect vitellogenins are large proteins (~200 kDa) synthesized in the fat body and processed in different ways depending on the insect groups that produce them. Unfortunately, IgE antibody binding to the full protein could not be tested because attempts to express the full-length vitellogenin from German cockroach (MW I18-N1862) resulted in a fragmented recombinant protein. Proteolytic cleavage of vitellogenin also occurs *in vivo* (21, 22). An N-terminal domain of vitellogenin (I18-S322-6xHis-tag) was also expressed for reasons explained above (**Supplemental Figure 1**). However, this fragment was not recognized by the subjects tested from our cohort (data not shown). Nevertheless, vitellogenin purified from natural source (German cockroach oothecae) has been reported to elicit specific IgE-mediated hypersensitivity responses, measured by intradermal skin test in Taiwan, and had a high IgE prevalence by immunoblot (46.88%; 15/32), following Bla g 2 and Bla g 4. Native vitellogenin comprised proteins of different molecular weights (97, 50, and 16 kDa) (15), consistent with protein fragmentation through cleavage sites reported in the vitellogenin sequence (19).

Bla g 3 was the second most dominant allergen for T cell activation after vitellogenin and was found to be a major allergen among highly cockroach sensitized subjects. Bla g 3 and the homolog Per a 3 are hexamerins, present in insect hemolymph and members of the hemocyanin super family. Hemocyanins are hexameric oxygen-carrying proteins (9, 11). The oligomerization of these allergens would increase its multivalency, and therefore its capacity to increase cross-linking of IgE in mast cells (39). However, for the purposes of measuring IgE as performed here, IgE produced against surface accessible epitopes in oligomeric Bla g 3 is expected to also react to the monomeric form, as mentioned above. In this study, IgE reacted to the recombinant allergen, indicating that, regardless of the oligomerization state, IgE epitopes are present in the recombinant protein tested for IgE antibody binding. Interestingly, Bla g 3 is present at higher amounts than other allergens in commercial cockroach extracts used for immunotherapy (29). Its concentration was 3, 10, 15, and 275-fold higher on average than that of Bla g 1, Bla g 2, Bla g 4, and Bla g 5, respectively, in 4 commercial extracts (29). Per a 3 was also abundant in extracts from body or feces of *P. americana* (45). The relative abundance of Bla g 3 in extracts might influence the effect of immunotherapy, and this allergen should therefore be considered when analyzing cockroach allergy clinical trials. Finally, the chitinase Bla g 12 did not display a high IgE prevalence, in contrast with reports for the homologs Per a 12 in China (63.8%), and Der p 15 (70%) and Der p 18 (63%) in Australia (12, 13). This is not surprising given the low amino acid identity between Bla g 12 and Per a 12 (34.1%) or Der p 15 (35.7%) or Der p 18 (27.0%).

In Taiwan, IgE-binding to Per a 2 was more frequently found among subjects with persistent asthma with allergic rhinitis (n = 21), than among individuals with rhinitis alone (n = 20) (81% versus 45%). On the other hand, 80% of allergic rhinitis patients had IgE-binding activity to Per a 9, versus only 28.5% of asthmatic patients. These results suggested that sensitization to Per a 2 could be a marker of more severe airway disease (46). However, in our study, no associations were observed between levels of cockroach and allergen-specific IgE antibody levels or allergen-specific T cell reactivity and disease phenotype/severity. CR-specific T cell responses have been characterized in relatively low detail (5–7, 47), and very little information is available particularly addressing if T cells play a role in cockroach allergic sensitization. Although it has been shown that higher T cell reactivity is associated with allergen sensitization and asthma (48), future studies are warranted to elucidate to which extent T cell responses correlate with clinical symptoms or disease severity. An important implication of the current study is that knowledge of individual reactivity profiles will help to interpret outcomes of cockroach immunotherapy, according to the allergens present in the cockroach extract used for treatment. A diagnostic analysis of each patient will provide information that could allow future tailored immunotherapy according to the proteins that are relevant for each patient.

## Data Availability Statement

The raw data supporting the conclusions of this article will be made available by the authors, without undue reservation.

## Ethics Statement

The studies involving human participants were reviewed and approved by Review boards: La Jolla Institute’s Institutional Review Board (IRB protocol: VD-112-0217), Mount Sinai’s Institutional Review Board (IRB protocol: GCO 13-0691), and Washington University Institutional Review Board (IRB protocol: 201305110). No vulnerable populations were involved. The patients/participants provided their written informed consent to participate in this study.

## Author Contributions

JG expressed and purified the new cockroach allergens and measured IgE reactivity. VS and RdSA planed the experimental T cell work and wrote the T cell section. ASu performed all the T cell focused experimental work. LBB, AB, and PB provided clinical samples. AF provided input for data analysis. AP and ASe designed the study. AP wrote the manuscript. All authors contributed to the article and approved the submitted version.

## Funding

Research reported in this publication was supported by the National Institute of Allergy And Infectious Diseases of the National Institutes of Health under Award Number R01AI077653 (to AP). The content is solely the responsibility of the authors and does not necessarily represent the official views of the National Institutes of Health.

## Conflict of Interest

AP is an employee of Indoor Biotechnologies, Inc. and the contact principal investigator of the NIH R01 Award that funded the study.

The remaining authors declare that the research was conducted in the absence of any commercial or financial relationships that could be construed as a potential conflict of interest.

## Abbreviations

PBS, phosphate buffered saline; WHO/IUIS, World Health Organization/International Union of Immunological Societies.
