# Supplementary material for: IgE and T Cell Reactivity to a Comprehensive Panel of Cockroach Allergens in Relation to Disease
Source: Front Immunol. 2021 Feb 10;11:621700. doi: 10.3389/fimmu.2020.621700 (PMC7902920; doi:10.3389/fimmu.2020.621700)
Supplement: Supplementary file 1 [file DataSheet_1.pdf]

Supplemental Figure

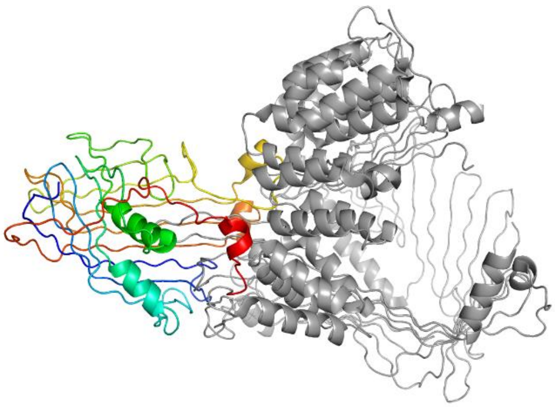

Supplemental Figure 1. X-ray crystal structure of lamprey lipovitellin (1lsh.pdb) (4) showing in color the N-sheet domain (up to residue Val 296), equivalent to the N-sheet from cockroach vitellogenin expressed in this study as N-vitellogenin.
