# Supplementary material for: IgE and T Cell Reactivity to a Comprehensive Panel of Cockroach Allergens in Relation to Disease
Source: Front Immunol. 2021 Feb 10;11:621700. doi: 10.3389/fimmu.2020.621700 (PMC7902920; doi:10.3389/fimmu.2020.621700)
Supplement: Supplementary file 2 [file DataSheet_2.pdf]

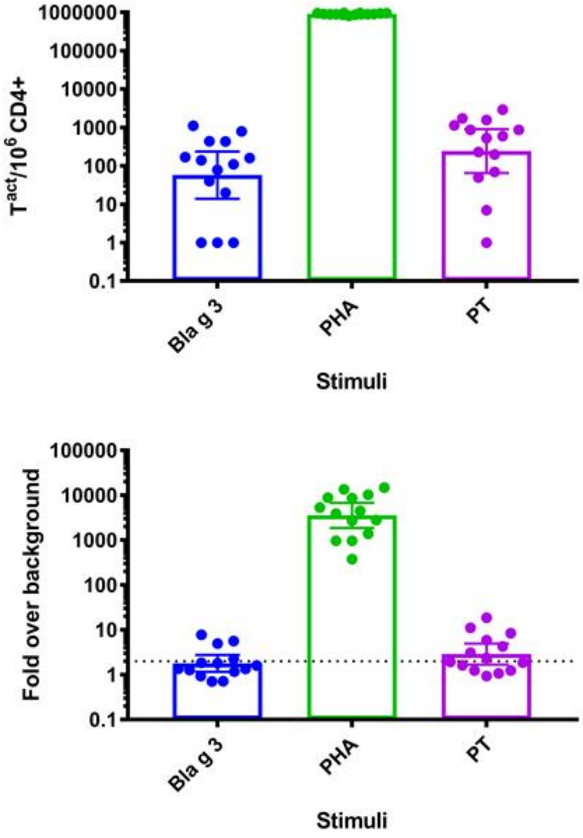

Supplemental Figure 2. Controls measured to assess allergen specific-T cell reactivity. PBMC were stimulated with cockroach allergen-derived peptide pools for 24 h. Subsequently, T cell activation (Ox40/PDL-1 expression) was measured by flow cytometry. Graph bars showing specific T cell reactivity for a representative allergen pool (Bla g 3), a polyclonal activator (PHA) or ubiquitous vaccine antigens pool (*Bordetella pertussis*, PT) expressed as absolute numbers (upper graph) or as fold increase over medium (lower graph). Each dot represents a donor. Geometric means with 95% CI are shown.
