# Supplementary material for: IgE and T Cell Reactivity to a Comprehensive Panel of Cockroach Allergens in Relation to Disease
Source: Front Immunol. 2021 Feb 10;11:621700. doi: 10.3389/fimmu.2020.621700 (PMC7902920; doi:10.3389/fimmu.2020.621700)
Supplement: Supplementary file 3 [file DataSheet_3.pdf]

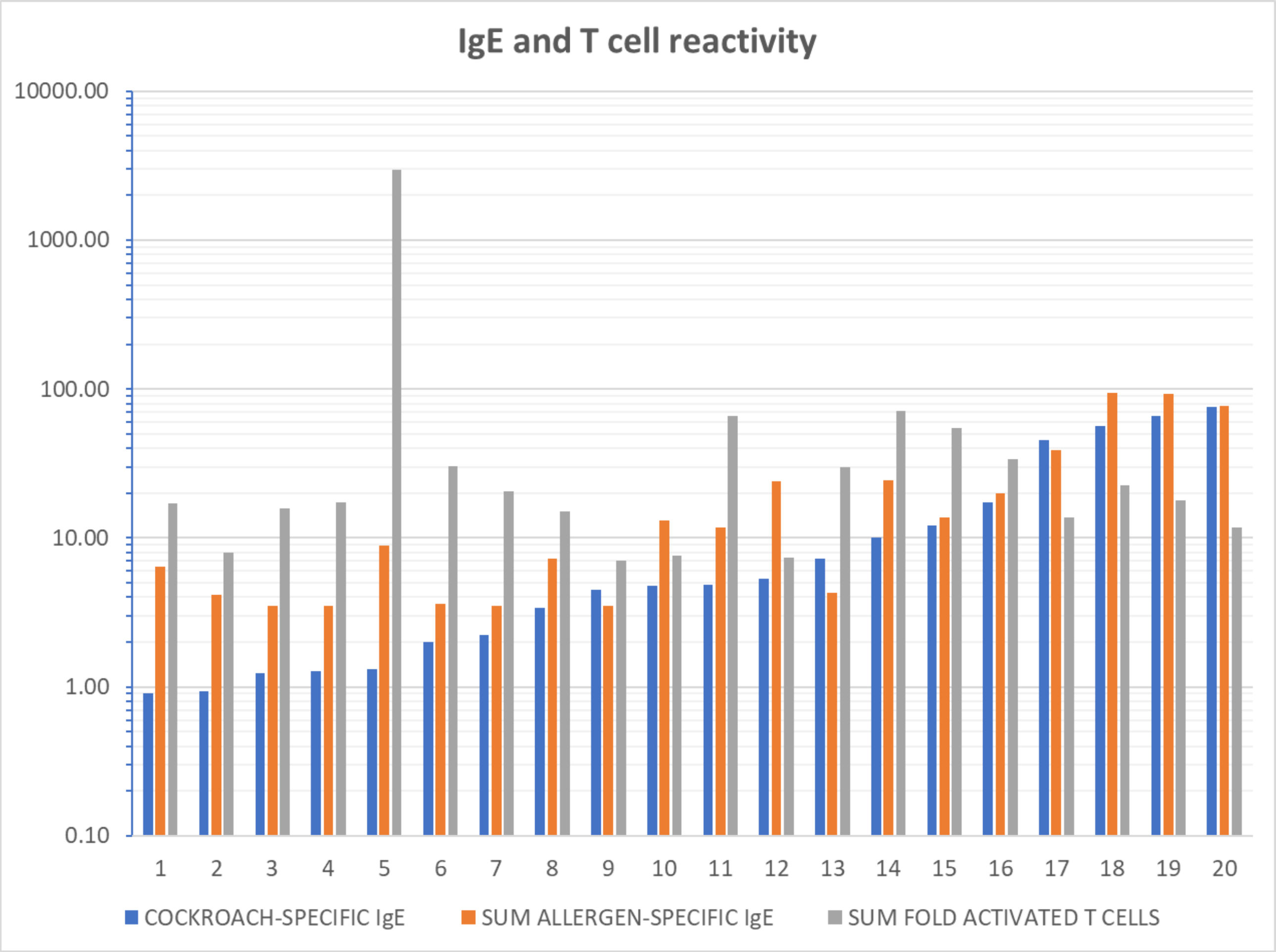

Supplemental Figure 3. All patients showed B and T cell reactivity to cockroach allergens (n = 14-20 donors tested for the 10 allergens). There was high correlation between cockroach-specific IgE and the sum of 10 allergen-specific IgE, but not between T and B cell reactivity. IgE antibody levels are shown in kU_A/L.
