# Supplementary material for: IgE and T Cell Reactivity to a Comprehensive Panel of Cockroach Allergens in Relation to Disease
Source: Front Immunol. 2021 Feb 10;11:621700. doi: 10.3389/fimmu.2020.621700 (PMC7902920; doi:10.3389/fimmu.2020.621700)
Supplement: Supplementary file 4 [file Table_1.pdf]

Supplemental Table 1. Sets of overlapping peptides from cockroach allergens used for T cell experiments

Allergen	start	end	peptide
Bla g 1	1	15	NLLEKLREKGVDVDK
Bla g 1	6	20	LREKGVDVDKIIELI
Bla g 1	11	25	VDVDKIIELIRALFG
Bla g 1	16	30	IIELIRALFGLTLNA
Bla g 1	21	35	RALFGLTLNAKASRN
Bla g 1	26	40	LTLNAKASRNLQDDL
Bla g 1	31	45	KASRNLQDDLQDFLA
Bla g 1	36	50	LQDDLQDFLALIPVD
Bla g 1	41	55	QDFLALIPVDQIIAI
Bla g 1	46	60	LIPVDQIIAIATDYL
Bla g 1	51	65	QIIAIATDYLANDAE
Bla g 1	56	70	ATDYLANDAEVQAAV
Bla g 1	61	75	ANDAEVQAAVAYLQS
Bla g 1	66	80	VQAAVAYLQSDEFET
Bla g 1	71	85	AYLQSDEFETIVVAL
Bla g 1	76	90	DEFETIVVALDALPE
Bla g 1	81	95	IVVALDALPELQNFL
Bla g 1	86	100	DALPELQNFLNFLEA
Bla g 1	91	105	LQNFLNFLEANGLNA
Bla g 1	96	110	NFLEANGLNAIDFLN
Bla g 1	101	115	NGLNAIDFLNGIHDL
Bla g 1	106	120	IDFLNGIHDLLGIPH
Bla g 1	111	125	GIHDLLGIPHIPVSG
Bla g 1	116	130	LGIPHIPVSGRKYHI
Bla g 1	121	135	IPVSGRKYHIRRGVG
Bla g 1	126	140	RKYHIRRGVGITGLI
Bla g 1	131	145	RRGVGITGLIDDVLA
Bla g 1	136	150	ITGLIDDVLAILPIE
Bla g 1	141	155	DDVLAILPIEDLKAL
Bla g 1	146	160	ILPIEDLKALFNEKL
Bla g 1	151	165	DLKALFNEKLETSPD
Bla g 1	156	170	FNEKLETSPDFLALY
Bla g 1	161	175	ETSPDFLALYNAIRS
Bla g 1	166	180	FLALYNAIRSPEFQS
Bla g 1	171	185	NAIRSPEFQSIVQTL
Bla g 1	176	190	PEFQSIVQTLNAMPE
Bla g 1	181	195	IVQTLNAMPEYQNLL
Bla g 1	186	200	NAMPEYQNLLQKLRE
Bla g 1	191	205	YQNLLQKLREKGVDV
Bla g 1	196	210	QKLREKGVDVDKIIE

Bla g 1	201	215	KGVDVDKIIELIRAL
Bla g 1	206	220	DKIIELIRALFGLTL
Bla g 1	211	225	LIRALFGLTLNGKAS
Bla g 1	216	230	FGLTLNGKASRNLQD
Bla g 1	221	235	NGKASRNLQDDLQDF
Bla g 1	226	240	RNLQDDLQDFLALIP
Bla g 1	231	245	DLQDFLALIPVDQII
Bla g 1	236	250	LALIPVDQIIAIATD
Bla g 1	241	255	VDQIIAIATDYLAND
Bla g 1	246	260	AIATDYLANDAEVQA
Bla g 1	251	265	YLANDAEVQAAVAYL
Bla g 1	256	270	AEVQAAVAYLQSDEF
Bla g 1	261	275	AVAYLQSDEFETIVV
Bla g 1	266	280	QSDEFETIVVTLDAL
Bla g 1	271	285	ETIVVTLDALPELQN
Bla g 1	276	290	TLDALPELQNFLNFL
Bla g 1	281	295	PELQNFLNFLEANGL
Bla g 1	286	300	FLNFLEANGLNAIDF
Bla g 1	291	305	EANGLNAIDFLNGIH
Bla g 1	296	310	NAIDFLNGIHDLLGI
Bla g 1	301	315	LNGIHDLLGIPHIPV
Bla g 1	306	320	DLLGIPHIPVSGRKY
Bla g 1	311	325	PHIPVSGRKYHIRRG
Bla g 1	316	330	SGRKYHIRRGVGITG
Bla g 1	321	335	HIRRGVGITGLIDDV
Bla g 1	326	340	VGITGLIDDVLAILP
Bla g 1	331	345	LIDDVLAILPLDDLK
Bla g 1	336	350	LAILPLDDLKALFNE
Bla g 1	341	355	LDDLKALFNEKLETS
Bla g 1	346	360	ALFNEKLETSPDFLA
Bla g 1	351	365	KLETSPDFLALYNAI
Bla g 1	356	370	PDFLALYNAIKSPEF
Bla g 1	361	375	LYNAIKSPEFQSIVQ
Bla g 1	366	380	KSPEFQSIVQTLNAM
Bla g 1	371	385	QSIVQTLNAMPEYQN
Bla g 1	376	390	TLNAMPEYQNLLEKL
Bla g 1	381	395	PEYQNLLEKLREKGV
Bla g 1	386	400	LLEKLREKGVDVDKI
Bla g 1	391	405	REKGVDVDKIIELIR
Bla g 1	396	410	DVDKIIELIRALFGL
Bla g 1	398	412	DKIIELIRALFGLTH
Bla g 2	1	15	VPLYKLVHVFINTQY
Bla g 2	6	20	LVHVFINTQYAGITK

Bla g 2	11	25	INTQYAGITKIGNQN
Bla g 2	16	30	AGITKIGNQNFLTVF
Bla g 2	21	35	IGNQNFLTVFDSTSC
Bla g 2	26	40	FLTVFDSTSCNVVVA
Bla g 2	31	45	DSTSCNVVVASQECV
Bla g 2	36	50	NVVVASQECVGGACV
Bla g 2	41	55	SQECVGGACVCPNLQ
Bla g 2	46	60	GGACVCPNLQKYEKL
Bla g 2	51	65	CPNLQKYEKLKPKYI
Bla g 2	56	70	KYEKLKPKYISDGNV
Bla g 2	61	75	KPKYISDGNVQVKFF
Bla g 2	66	80	SDGNVQVKFFDTGSA
Bla g 2	71	85	QVKFFDTGSAVGRGI
Bla g 2	76	90	DTGSAVGRGIEDSLT
Bla g 2	81	95	VGRGIEDSLTISQLT
Bla g 2	86	100	EDSLTISQLTTSQQD
Bla g 2	91	105	ISQLTTSQQDIVLAD
Bla g 2	96	110	TSQQDIVLADELSQE
Bla g 2	101	115	IVLADELSQEVCILS
Bla g 2	106	120	ELSQEVCILSADVVV
Bla g 2	111	125	VCILSADVVVGIAAP
Bla g 2	116	130	ADVVVGIAAPGCPNA
Bla g 2	121	135	GIAAPGCPNALKGKT
Bla g 2	126	140	GCPNALKGKTVLENF
Bla g 2	131	145	LKGKTVLENFVEENL
Bla g 2	136	150	VLENFVEENLIAPVF
Bla g 2	141	155	VEENLIAPVFSIHHA
Bla g 2	146	160	IAPVFSIHHARFQDG
Bla g 2	151	165	SIHHARFQDGEHFGE
Bla g 2	156	170	RFQDGEHFGEIIFGG
Bla g 2	161	175	EHFGEIIFGGSDWKY
Bla g 2	166	180	IIFGGSDWKYVDGEF
Bla g 2	171	185	SDWKYVDGEFTYVPL
Bla g 2	176	190	VDGEFTYVPLVGDDS
Bla g 2	181	195	TYVPLVGDDSWKFRL
Bla g 2	186	200	VGDDSWKFRLDGVKI
Bla g 2	191	205	WKFRLDGVKIGDTTV
Bla g 2	196	210	DGVKIGDTTVAPAGT
Bla g 2	201	215	GDTTVAPAGTQAIID
Bla g 2	206	220	APAGTQAIIDTSKAI
Bla g 2	211	225	QAIIDTSKAIIVGPK
Bla g 2	216	230	TSKAIIVGPKAYVNP
Bla g 2	221	235	IVGPKAYVNPINEAI

Bla g 2	226	240	AYVNPINEAIGCVVE
Bla g 2	231	245	INEAIGCVVEKTTTR
Bla g 2	236	250	GCVVEKTTTRRICKL
Bla g 2	241	255	KTTTRRICKLDCSKI
Bla g 2	246	260	RICKLDCSKIPSLPD
Bla g 2	251	265	DCSKIPSLPDVTFVI
Bla g 2	256	270	PSLPDVTFVINGRNF
Bla g 2	261	275	VTFVINGRNFNISSQ
Bla g 2	266	280	NGRNFNISSQYYIQQ
Bla g 2	271	285	NISSQYYIQQNGNLC
Bla g 2	276	290	YYIQQNGNLCYSGFQ
Bla g 2	281	295	NGNLCYSGFQPCGHS
Bla g 2	286	300	YSGFQPCGHSDHFFI
Bla g 2	291	305	PCGHSDHFFIGDFFV
Bla g 2	296	310	DHFFIGDFFVDHYYS
Bla g 2	301	315	GDFFVDHYYSEFNWE
Bla g 2	306	320	DHYYSEFNWENKTMG
Bla g 2	311	325	EFNWENKTMGFGRSV
Bla g 2	314	328	WENKTMGFGRSVESV
Bla g 4	1	15	NEDCFRHESLVPNLD
Bla g 4	6	20	RHESLVPNLDYERFR
Bla g 4	11	25	VPNLDYERFRGSWII
Bla g 4	16	30	YERFRGSWIIAAGTS
Bla g 4	21	35	GSWIIAAGTSEALTQ
Bla g 4	26	40	AAGTSEALTQYKCWI
Bla g 4	31	45	EALTQYKCWIDRFSY
Bla g 4	36	50	YKCWIDRFSYDDALV
Bla g 4	41	55	DRFSYDDALVSKYTD
Bla g 4	46	60	DDALVSKYTDSQGKN
Bla g 4	51	65	SKYTDSQGKNRTTIR
Bla g 4	56	70	SQGKNRTTIRGRTKF
Bla g 4	61	75	RTTIRGRTKFEGNKF
Bla g 4	66	80	GRTKFEGNKFTIDYN
Bla g 4	71	85	EGNKFTIDYNDKGKA
Bla g 4	76	90	TIDYNDKGKAFSAPY
Bla g 4	81	95	DKGKAFSAPYSVLAT
Bla g 4	86	100	FSAPYSVLATDYENY
Bla g 4	91	105	SVLATDYENYAIVEG
Bla g 4	96	110	DYENYAIVEGCPAAA
Bla g 4	101	115	AIVEGCPAAANGHVI
Bla g 4	106	120	CPAAANGHVIYVQIR
Bla g 4	111	125	NGHVIYVQIRFSVRR
Bla g 4	116	130	YVQIRFSVRRFHPKL

Bla g 4	121	135	FSVRRFHPKLGDKEM
Bla g 4	126	140	FHPKLGDKEMIQHYT
Bla g 4	131	145	GDKEMIQHYTLDQVN
Bla g 4	136	150	IQHYTLDQVNQHKKA
Bla g 4	141	155	LDQVNQHKKAIEEDL
Bla g 4	146	160	QHKKAIEEDLKHFNL
Bla g 4	151	165	IEEDLKHFNLKYEDL
Bla g 4	156	170	KHFNLKYEDLHSTCH
Bla g 5	1	15	APSYKLTYCPVKALG
Bla g 5	6	20	LTYCPVKALGEPIRF
Bla g 5	11	25	VKALGEPIRFLLSYG
Bla g 5	16	30	EPIRFLLSYGEKDFE
Bla g 5	21	35	LLSYGEKDFEDYRFQ
Bla g 5	26	40	EKDFEDYRFQEGDWP
Bla g 5	31	45	DYRFQEGDWPNLKPS
Bla g 5	36	50	EGDWPNLKPSMPFGK
Bla g 5	41	55	NLKPSMPFGKTPVLE
Bla g 5	46	60	MPFGKTPVLEIDGKQ
Bla g 5	51	65	TPVLEIDGKQTHQSV
Bla g 5	56	70	IDGKQTHQSVAISRY
Bla g 5	61	75	THQSVAISRYLGKQF
Bla g 5	66	80	AISRYLGKQFGLSGK
Bla g 5	71	85	LGKQFGLSGKDDWEN
Bla g 5	76	90	GLSGKDDWENLEIDM
Bla g 5	81	95	DDWENLEIDMIVDTI
Bla g 5	86	100	LEIDMIVDTISDFRA
Bla g 5	91	105	IVDTISDFRAAIANY
Bla g 5	96	110	SDFRAAIANYHYDAD
Bla g 5	101	115	AIANYHYDADENSKQ
Bla g 5	106	120	HYDADENSKQKKWDP
Bla g 5	111	125	ENSKQKKWDPLKKET
Bla g 5	116	130	KKWDPLKKETIPYYT
Bla g 5	121	135	LKKETIPYYTKKFDE
Bla g 5	126	140	IPYYTKKFDEVVKAN
Bla g 5	131	145	KKFDEVVKANGGYLA
Bla g 5	136	150	VVKANGGYLAAGKLT
Bla g 5	141	155	GGYLAAGKLTWADFY
Bla g 5	146	160	AGKLTWADFYFVAIL
Bla g 5	151	165	WADFYFVAILDYLNH
Bla g 5	156	170	FVAILDYLNHMAKED
Bla g 5	161	175	DYLNHMAKEDLVANQ
Bla g 5	166	180	MAKEDLVANQPNLKA
Bla g 5	171	185	LVANQPNLKALREKV

Bla g 5	176	190	PNLKALREKVLGLPA
Bla g 5	181	195	LREKVLGLPAIKAWV
Bla g 5	186	200	LGLPAIKAWVAKRPP
Bla g 5	189	203	PAIKAWVAKRPPTDL
Bla g 6	1	15	MDELPPEQIQLLKKA
Bla g 6	6	20	PEQIQLLKKAFDAFD
Bla g 6	11	25	LLKKAFDAFDREKKG
Bla g 6	16	30	FDAFDREKKGCISTE
Bla g 6	21	35	REKKGCISTEMVGTI
Bla g 6	26	40	CISTEMVGTILEMLG
Bla g 6	31	45	MVGTILEMLGHRLDD
Bla g 6	36	50	LEMLGHRLDDDMLQE
Bla g 6	41	55	HRLDDDMLQEIIAEV
Bla g 6	46	60	DMLQEIIAEVDADGS
Bla g 6	51	65	IIAEVDADGSGELEF
Bla g 6	56	70	DADGSGELEFEEFVS
Bla g 6	61	75	GELEFEEFVSLASRF
Bla g 6	66	80	EEFVSLASRFLVEED
Bla g 6	71	85	LASRFLVEEDAEAMQ
Bla g 6	76	90	LVEEDAEAMQQELRE
Bla g 6	81	95	AEAMQQELREAFRLY
Bla g 6	86	100	QELREAFRLYDKEGN
Bla g 6	91	105	AFRLYDKEGNGYITT
Bla g 6	96	110	DKEGNGYITTNVLRE
Bla g 6	101	115	GYITTNVLREILKEL
Bla g 6	106	120	NVLREILKELDDKIT
Bla g 6	111	125	ILKELDDKITAEDLD
Bla g 6	116	130	DDKITAEDLDMMIEE
Bla g 6	121	135	AEDLDMMIEEIDSDG
Bla g 6	126	140	MMIEEIDSDGSGTVD
Bla g 6	131	145	IDSDGSGTVDFDEFM
Bla g 6	136	150	SGTVDFDEFMEVMTG
Bla g 6	137	151	GTVDFDEFMEVMTGE
Per a 7	1	15	MDAIKKKMQAMKLEK
Per a 7	6	20	KKMQAMKLEKDNAMD
Per a 7	11	25	MKLEKDNAMDRALLC
Per a 7	16	30	DNAMDRALLCEQQAR
Per a 7	21	35	RALLCEQQARDANLR
Per a 7	26	40	EQQARDANLRAEKAE
Per a 7	31	45	DANLRAEKAEEEARS
Per a 7	36	50	AEKAEEEARSLQKKI
Per a 7	41	55	EEARSLQKKIQQIEN
Per a 7	46	60	LQKKIQQIENDLDQT

Per a 7	51	65	QQIENDLDQTMEQLM
Per a 7	56	70	DLDQTMEQLMQVNAK
Per a 7	61	75	MEQLMQVNAKLDEKD
Per a 7	66	80	QVNAKLDEKDKALQN
Per a 7	71	85	LDEKDKALQNAESEV
Per a 7	76	90	KALQNAESEVAALNR
Per a 7	81	95	AESEVAALNRRIQLL
Per a 7	86	100	AALNRRIQLLEEDLE
Per a 7	91	105	RIQLLEEDLERSEER
Per a 7	96	110	EEDLERSEERLATAT
Per a 7	101	115	RSEERLATATAKLAE
Per a 7	106	120	LATATAKLAEASQAA
Per a 7	111	125	AKLAEASQAADESER
Per a 7	116	130	ASQAADESERARKIL
Per a 7	121	135	DESERARKILESKGL
Per a 7	126	140	ARKILESKGLADEER
Per a 7	131	145	ESKGLADEERMDALE
Per a 7	136	150	ADEERMDALENQLKE
Per a 7	141	155	MDALENQLKEARFMA
Per a 7	146	160	NQLKEARFMAEEADK
Per a 7	151	165	ARFMAEEADKKYDEV
Per a 7	156	170	EEADKKYDEVARKLA
Per a 7	161	175	KYDEVARKLAMVEAD
Per a 7	166	180	ARKLAMVEADLERAE
Per a 7	171	185	MVEADLERAEERAES
Per a 7	176	190	LERAEERAESGESKI
Per a 7	181	195	ERAESGESKIVELEE
Per a 7	186	200	GESKIVELEEELRVV
Per a 7	191	205	VELEEELRVVGNNLK
Per a 7	196	210	ELRVVGNNLKSLEVS
Per a 7	201	215	GNNLKSLEVSEEKAN
Per a 7	206	220	SLEVSEEKANLREEE
Per a 7	211	225	EEKANLREEEYKQQI
Per a 7	216	230	LREEEYKQQIKTLTT
Per a 7	221	235	YKQQIKTLTTRLKEA
Per a 7	226	240	KTLTTRLKEAEARAE
Per a 7	231	245	RLKEAEARAEFAERS
Per a 7	236	250	EARAEFAERSVQKLQ
Per a 7	241	255	FAERSVQKLQKEVDR
Per a 7	246	260	VQKLQKEVDRLEDEL
Per a 7	251	265	KEVDRLEDELVHEKE
Per a 7	256	270	LEDELVHEKEKYKFI
Per a 7	261	275	VHEKEKYKFICDDLD

Per a 7	266	280	KYKFICDDLDMTFTE
Per a 7	270	284	ICDDLDMTFTELIGN
Bla g 9	1	15	MVDAAVLEKLEAGFA
Bla g 9	6	20	VLEKLEAGFAKLAAS
Bla g 9	11	25	EAGFAKLAASDSKSL
Bla g 9	16	30	KLAASDSKSLLRKYL
Bla g 9	21	35	DSKSLLRKYLTKEVF
Bla g 9	26	40	LRKYLTKEVFDNLKT
Bla g 9	31	45	TKEVFDNLKTKKTPT
Bla g 9	36	50	DNLKTKKTPTFGSTL
Bla g 9	41	55	KKTPTFGSTLLDVIQ
Bla g 9	46	60	FGSTLLDVIQSGLEN
Bla g 9	51	65	LDVIQSGLENHDSGV
Bla g 9	56	70	SGLENHDSGVGIYAP
Bla g 9	61	75	HDSGVGIYAPDAEAY
Bla g 9	66	80	GIYAPDAEAYTVFAD
Bla g 9	71	85	DAEAYTVFADLFDPI
Bla g 9	76	90	TVFADLFDPIIEDYH
Bla g 9	81	95	LFDPIIEDYHGGFKK
Bla g 9	86	100	IEDYHGGFKKTDKHP
Bla g 9	91	105	GGFKKTDKHPPKDWG
Bla g 9	96	110	TDKHPPKDWGDVDTL
Bla g 9	101	115	PKDWGDVDTLGNLDP
Bla g 9	106	120	DVDTLGNLDPAGEYI
Bla g 9	111	125	GNLDPAGEYIISTRV
Bla g 9	116	130	AGEYIISTRVRCGRS
Bla g 9	121	135	ISTRVRCGRSMQGYP
Bla g 9	126	140	RCGRSMQGYPFNPCL
Bla g 9	131	145	MQGYPFNPCLTEAQY
Bla g 9	136	150	FNPCLTEAQYKEMED
Bla g 9	141	155	TEAQYKEMEDKVSST
Bla g 9	146	160	KEMEDKVSSTLSGLE
Bla g 9	151	165	KVSSTLSGLEGELKG
Bla g 9	156	170	LSGLEGELKGQFYPL
Bla g 9	161	175	GELKGQFYPLTGMTK
Bla g 9	166	180	QFYPLTGMTKEVQQK
Bla g 9	171	185	TGMTKEVQQKLIDDH
Bla g 9	176	190	EVQQKLIDDHFLFKE
Bla g 9	181	195	LIDDHFLFKEGDRFL
Bla g 9	186	200	FLFKEGDRFLQHANA
Bla g 9	191	205	GDRFLQHANACRFWP
Bla g 9	196	210	QHANACRFWPTGRGI
Bla g 9	201	215	CRFWPTGRGIYHNDA

Bla g 9	206	220	TGRGIYHNDAKTFLV
Bla g 9	211	225	YHNDAKTFLVWCNEE
Bla g 9	216	230	KTFLVWCNEEDHLRI
Bla g 9	221	235	WCNEEDHLRIISMQM
Bla g 9	226	240	DHLRIISMQMGGDLG
Bla g 9	231	245	ISMQMGGDLGQVYRR
Bla g 9	236	250	GGDLGQVYRRLVTAV
Bla g 9	241	255	QVYRRLVTAVNDIEK
Bla g 9	246	260	LVTAVNDIEKRVPFS
Bla g 9	251	265	NDIEKRVPFSHDDRL
Bla g 9	256	270	RVPFSHDDRLGFLTF
Bla g 9	261	275	HDDRLGFLTFCPTNL
Bla g 9	266	280	GFLTFCPTNLGTTVR
Bla g 9	271	285	CPTNLGTTVRASVRI
Bla g 9	276	290	GTTVRASVRIKVPKL
Bla g 9	281	295	ASVRIKVPKLAADKK
Bla g 9	286	300	KVPKLAADKKKLEEV
Bla g 9	291	305	AADKKKLEEVAGKYN
Bla g 9	296	310	KLEEVAGKYNLQVRG
Bla g 9	301	315	AGKYNLQVRGTRGEH
Bla g 9	306	320	LQVRGTRGEHTEAEG
Bla g 9	311	325	TRGEHTEAEGGVYDI
Bla g 9	316	330	TEAEGGVYDISNKRR
Bla g 9	321	335	GVYDISNKRRMGLTE
Bla g 9	326	340	SNKRRMGLTEYDAVK
Bla g 9	331	345	MGLTEYDAVKGMNDG
Bla g 9	336	350	YDAVKGMNDGIAELI
Bla g 9	341	355	GMNDGIAELIKIESS
Bla g 9	342	356	MNDGIAELIKIESSL
Bla g 11	1	15	MKLFPLVALLVLVVG
Bla g 11	6	20	LVALLVLVVGVLSQK
Bla g 11	11	25	VLVVGVLSQKDPHVW
Bla g 11	16	30	VLSQKDPHVWDGRSA
Bla g 11	21	35	DPHVWDGRSAIVHLF
Bla g 11	26	40	DGRSAIVHLFEWKFA
Bla g 11	31	45	IVHLFEWKFADIADE
Bla g 11	36	50	EWKFADIADECERFL
Bla g 11	41	55	DIADECERFLGPKGF
Bla g 11	46	60	CERFLGPKGFAGVQV
Bla g 11	51	65	GPKGFAGVQVSPVHE
Bla g 11	56	70	AGVQVSPVHENVIIS
Bla g 11	61	75	SPVHENVIISSPFRP
Bla g 11	66	80	NVIISSPFRPWWERY

Bla g 11	71	85	SPFRPWWERYQLVSY
Bla g 11	76	90	WWERYQLVSYKLVSR
Bla g 11	81	95	QLVSYKLVSRSGDEN
Bla g 11	86	100	KLVSRSGDENAFRDM
Bla g 11	91	105	SGDENAFRDMVRRCN
Bla g 11	96	110	AFRDMVRRCNNVGIR
Bla g 11	101	115	VRRCNNVGIRIYVDV
Bla g 11	106	120	NVGIRIYVDVVLNQM
Bla g 11	111	125	IYVDVVLNQMSGSWP
Bla g 11	116	130	VLNQMSGSWPDAHGQ
Bla g 11	121	135	SGSWPDAHGQGGSTA
Bla g 11	126	140	DAHGQGGSTADTYNL
Bla g 11	131	145	GGSTADTYNLQYPAV
Bla g 11	136	150	DTYNLQYPAVPYGPG
Bla g 11	141	155	QYPAVPYGPGDFHST
Bla g 11	146	160	PYGPGDFHSTCTVSN
Bla g 11	151	165	DFHSTCTVSNYQDPS
Bla g 11	156	170	CTVSNYQDPSNVRNC
Bla g 11	161	175	YQDPSNVRNCELVGL
Bla g 11	166	180	NVRNCELVGLHDLNQ
Bla g 11	171	185	ELVGLHDLNQGSDYV
Bla g 11	176	190	HDLNQGSDYVRGKMI
Bla g 11	181	195	GSDYVRGKMIEYLNH
Bla g 11	186	200	RGKMIEYLNHLVDCG
Bla g 11	191	205	EYLNHLVDCGVAGFR
Bla g 11	196	210	LVDCGVAGFRVDAAK
Bla g 11	201	215	VAGFRVDAAKHMWPA
Bla g 11	206	220	VDAAKHMWPADLQYI
Bla g 11	211	225	HMWPADLQYIYSKVN
Bla g 11	216	230	DLQYIYSKVNNLNTD
Bla g 11	221	235	YSKVNNLNTDHGFPS
Bla g 11	226	240	NLNTDHGFPSGARPF
Bla g 11	231	245	HGFPSGARPFFYQEV
Bla g 11	236	250	GARPFFYQEVIDLGG
Bla g 11	241	255	FYQEVIDLGGEAIHS
Bla g 11	246	260	IDLGGEAIHSTEYTG
Bla g 11	251	265	EAIHSTEYTGFGRVT
Bla g 11	256	270	TEYTGFGRVTEFKYS
Bla g 11	261	275	FGRVTEFKYSRDIGD
Bla g 11	266	280	EFKYSRDIGDAFRGN
Bla g 11	271	285	RDIGDAFRGNNAIKW
Bla g 11	276	290	AFRGNNAIKWLVNFG
Bla g 11	281	295	NAIKWLVNFGVGWGY

Bla g 11	286	300	LVNFGVGWGYIPDGD
Bla g 11	291	305	VGWGYIPDGDALVFV
Bla g 11	296	310	IPDGDALVFVDNHDN
Bla g 11	301	315	ALVFVDNHDNQRGHG
Bla g 11	306	320	DNHDNQRGHGAGGAS
Bla g 11	311	325	QRGHGAGGASILTYK
Bla g 11	316	330	AGGASILTYKTSKLY
Bla g 11	321	335	ILTYKTSKLYKMAVA
Bla g 11	326	340	TSKLYKMAVAFMLAY
Bla g 11	331	345	KMAVAFMLAYPYGYP
Bla g 11	336	350	FMLAYPYGYPRVMSS
Bla g 11	341	355	PYGYPRVMSSFSFDN
Bla g 11	346	360	RVMSSFSFDNSDQGP
Bla g 11	351	365	FSFDNSDQGPPQDGN
Bla g 11	356	370	SDQGPPQDGNGNIIS
Bla g 11	361	375	PQDGNGNIISPSINA
Bla g 11	366	380	GNIISPSINADGTCG
Bla g 11	371	385	PSINADGTCGNGWVC
Bla g 11	376	390	DGTCGNGWVCEHRWR
Bla g 11	381	395	NGWVCEHRWRQIFNM
Bla g 11	386	400	EHRWRQIFNMVGFRN
Bla g 11	391	405	QIFNMVGFRNAVAGT
Bla g 11	396	410	VGFRNAVAGTAVSNW
Bla g 11	401	415	AVAGTAVSNWWDNGD
Bla g 11	406	420	AVSNWWDNGDKQISF
Bla g 11	411	425	WDNGDKQISFCRGNK
Bla g 11	416	430	KQISFCRGNKGFVAF
Bla g 11	421	435	CRGNKGFVAFNDEFN
Bla g 11	426	440	GFVAFNDEFNNDLKQ
Bla g 11	431	445	NDEFNNDLKQTLQTC
Bla g 11	436	450	NDLKQTLQTCLPAGD
Bla g 11	441	455	TLQTCLPAGDYCDVI
Bla g 11	446	460	LPAGDYCDVISGSYE
Bla g 11	451	465	YCDVISGSYENGSCT
Bla g 11	456	470	SGSYENGSCTGKTVT
Bla g 11	461	475	NGSCTGKTVTVGSDG
Bla g 11	466	480	GKTVTVGSDGKAYIE
Bla g 11	471	485	VGSDGKAYIEILSSA
Bla g 11	476	490	KAYIEILSSADDGVL
Bla g 11	481	495	ILSSADDGVLAIHVN
Bla g 11	486	500	DDGVLAIHVNSKVGS
Bla g 11	491	505	AIHVNSKVGSKSQTT
Bla g 11	496	510	SKVGSKSQTTTTQSS

Bla g 11	501	515	KSQTTTTQSSHCTCS
Bla g 3	1	15	IPADKVFLRKQWDVL
Bla g 3	6	20	VFLRKQWDVLRLVHR
Bla g 3	11	25	QWDVLRLVHRIHQHS
Bla g 3	16	30	RLVHRIHQHSIIPEQ
Bla g 3	21	35	IHQHSIIPEQVTVGD
Bla g 3	26	40	IIPEQVTVGDSYDIE
Bla g 3	31	45	VTVGDSYDIEANINN
Bla g 3	36	50	SYDIEANINNYKNPR
Bla g 3	41	55	ANINNYKNPRVVKNF
Bla g 3	46	60	YKNPRVVKNFMALYK
Bla g 3	51	65	VVKNFMALYKKDPVK
Bla g 3	56	70	MALYKKDPVKRGEPF
Bla g 3	61	75	KDPVKRGEPFSTYYI
Bla g 3	66	80	RGEPFSTYYIKHREQ
Bla g 3	71	85	STYYIKHREQAIMLF
Bla g 3	76	90	KHREQAIMLFELFYY
Bla g 3	81	95	AIMLFELFYYANDYD
Bla g 3	86	100	ELFYYANDYDTFYKT
Bla g 3	91	105	ANDYDTFYKTACWAR
Bla g 3	96	110	TFYKTACWARDRVNE
Bla g 3	101	115	ACWARDRVNEGMFLY
Bla g 3	106	120	DRVNEGMFLYSFNIA
Bla g 3	111	125	GMFLYSFNIAIMHRE
Bla g 3	116	130	SFNIAIMHREDMQDI
Bla g 3	121	135	IMHREDMQDIVVPAF
Bla g 3	126	140	DMQDIVVPAFYEIYP
Bla g 3	131	145	VVPAFYEIYPFLFVE
Bla g 3	136	150	YEIYPFLFVENDVIQ
Bla g 3	141	155	FLFVENDVIQKAYDY
Bla g 3	146	160	NDVIQKAYDYKMKES
Bla g 3	151	165	KAYDYKMKESGHLNE
Bla g 3	156	170	KMKESGHLNEPHTHV
Bla g 3	161	175	GHLNEPHTHVIPVNF
Bla g 3	166	180	PHTHVIPVNFTLRNQ
Bla g 3	171	185	IPVNFTLRNQEQLLS
Bla g 3	176	190	TLRNQEQLLSYFTED
Bla g 3	181	195	EQLLSYFTEDVFLNA
Bla g 3	186	200	YFTEDVFLNAFNTYF
Bla g 3	191	205	VFLNAFNTYFRYMYP
Bla g 3	196	210	FNTYFRYMYPTWFNY
Bla g 3	201	215	RYMYPTWFNYTKYEY
Bla g 3	206	220	TWFNYTKYEYDVPRH

Bla g 3	211	225	TKYEYDVPRHGEQFY
Bla g 3	216	230	DVPRHGEQFYYFNQQ
Bla g 3	221	235	GEQFYYFNQQMFARY
Bla g 3	226	240	YFNQQMFARYMLERY
Bla g 3	231	245	MFARYMLERYSNDMP
Bla g 3	236	250	MLERYSNDMPEIQPF
Bla g 3	241	255	SNDMPEIQPFTYTKP
Bla g 3	246	260	EIQPFTYTKPFKTPY
Bla g 3	251	265	TYTKPFKTPYNPQLR
Bla g 3	256	270	FKTPYNPQLRYPNGQ
Bla g 3	261	275	NPQLRYPNGQEVPAR
Bla g 3	266	280	YPNGQEVPARPAYMM
Bla g 3	271	285	EVPARPAYMMPQDFD
Bla g 3	276	290	PAYMMPQDFDLMYVS
Bla g 3	281	295	PQDFDLMYVSDIKNY
Bla g 3	286	300	LMYVSDIKNYEKRVA
Bla g 3	291	305	DIKNYEKRVADAVDF
Bla g 3	296	310	EKRVADAVDFGYVFC
Bla g 3	301	315	DAVDFGYVFCDKMIS
Bla g 3	306	320	GYVFCDKMISHSLYN
Bla g 3	311	325	DKMISHSLYNNEKGL
Bla g 3	316	330	HSLYNNEKGLEWLGQ
Bla g 3	321	335	NEKGLEWLGQIVEGN
Bla g 3	326	340	EWLGQIVEGNSMHPD
Bla g 3	331	345	IVEGNSMHPDFYGHI
Bla g 3	336	350	SMHPDFYGHIFHMYR
Bla g 3	341	355	FYGHIFHMYRSLLGH
Bla g 3	346	360	FHMYRSLLGHITDPF
Bla g 3	351	365	SLLGHITDPFHKHGV
Bla g 3	356	370	ITDPFHKHGVAPSAL
Bla g 3	361	375	HKHGVAPSALEHPET
Bla g 3	366	380	APSALEHPETSLRDP
Bla g 3	371	385	EHPETSLRDPAFYQI
Bla g 3	376	390	SLRDPAFYQIWKRVQ
Bla g 3	381	395	AFYQIWKRVQHYFNK
Bla g 3	386	400	WKRVQHYFNKFQMKQ
Bla g 3	391	405	HYFNKFQMKQPYYTR
Bla g 3	396	410	FQMKQPYYTREELAF
Bla g 3	401	415	PYYTREELAFDGVKI
Bla g 3	406	420	EELAFDGVKIDNVDV
Bla g 3	411	425	DGVKIDNVDVGKLYT
Bla g 3	416	430	DNVDVGKLYTYFEPY
Bla g 3	421	435	GKLYTYFEPYEMGLS

Bla g 3	426	440	YFEPYEMGLSNAVKV
Bla g 3	431	445	EMGLSNAVKVGKLED
Bla g 3	436	450	NAVKVGKLEDVPNVD
Bla g 3	441	455	GKLEDVPNVDIRARN
Bla g 3	446	460	VPNVDIRARNYRLNH
Bla g 3	451	465	IRARNYRLNHKPFTY
Bla g 3	456	470	YRLNHKPFTYNVEVT
Bla g 3	461	475	KPFTYNVEVTSEKDT
Bla g 3	466	480	NVEVTSEKDTPVYVR
Bla g 3	471	485	SEKDTPVYVRVFLGP
Bla g 3	476	490	PVYVRVFLGPKYNYY
Bla g 3	481	495	VFLGPKYNYYGHEYD
Bla g 3	486	500	KYNYYGHEYDLNERR
Bla g 3	491	505	GHEYDLNERRNYFVE
Bla g 3	496	510	LNERRNYFVEIDRFP
Bla g 3	501	515	NYFVEIDRFPYQLHT
Bla g 3	506	520	IDRFPYQLHTGKNTI
Bla g 3	511	525	YQLHTGKNTIQRNSH
Bla g 3	516	530	GKNTIQRNSHDSSVV
Bla g 3	521	535	QRNSHDSSVVAQEQD
Bla g 3	526	540	DSSVVAQEQDTYKVL
Bla g 3	531	545	AQEQDTYKVLYKKVN
Bla g 3	536	550	TYKVLYKKVNEAYEG
Bla g 3	541	555	YKKVNEAYEGKTTYT
Bla g 3	546	560	EAYEGKTTYTYEKQD
Bla g 3	551	565	KTTYTYEKQDKYCGL
Bla g 3	556	570	YEKQDKYCGLPEHLL
Bla g 3	561	575	KYCGLPEHLLIPKGK
Bla g 3	566	580	PEHLLIPKGKKGGQA
Bla g 3	571	585	IPKGKKGGQAFTVYV
Bla g 3	576	590	KGGQAFTVYVIVTPY
Bla g 3	581	595	FTVYVIVTPYDKAVE
Bla g 3	586	600	IVTPYDKAVEKEEHH
Bla g 3	591	605	DKAVEKEEHHFKAYS
Bla g 3	596	610	KEEHHFKAYSYCGVG
Bla g 3	601	615	FKAYSYCGVGPHDSV
Bla g 3	606	620	YCGVGPHDSVYDKKP
Bla g 3	611	625	PHDSVYDKKPLGFPF
Bla g 3	616	630	YDKKPLGFPFDRPIH
Bla g 3	621	635	LGFPFDRPIHSYDFV
Bla g 3	626	640	DRPIHSYDFVTPNMF
Bla g 3	631	645	SYDFVTPNMFMKDVF
Bla g 3	636	650	TPNMFMKDVFIFHKK

Bla g 3	641	655	MKDVFIFHKKYEEVE
Bla g 3	643	660	DVFIFHKKYEEVEQH
chitinase	1	15	DKPSRVVCYFSNWAV
chitinase	6	20	VVCYFSNWAVYRPGL
chitinase	11	25	SNWAVYRPGLGSYKI
chitinase	16	30	YRPGLGSYKIEDIPT
chitinase	21	35	GSYKIEDIPTDLCTH
chitinase	26	40	EDIPTDLCTHLIYSF
chitinase	31	45	DLCTHLIYSFIGVSN
chitinase	36	50	LIYSFIGVSNVTWGP
chitinase	41	55	IGVSNVTWGPLILDQ
chitinase	46	60	VTWGPLILDQENDVD
chitinase	51	65	LILDQENDVDLRGFL
chitinase	56	70	ENDVDLRGFLNFTDL
chitinase	61	75	LRGFLNFTDLKAKGV
chitinase	66	80	NFTDLKAKGVKTSVA
chitinase	71	85	KAKGVKTSVAMGGWG
chitinase	76	90	KTSVAMGGWGEGGRK
chitinase	81	95	MGGWGEGGRKYSHLV
chitinase	86	100	EGGRKYSHLVSDKKL
chitinase	91	105	YSHLVSDKKLRDTFI
chitinase	96	110	SDKKLRDTFIPALVE
chitinase	101	115	RDTFIPALVEFLHKY
chitinase	106	120	PALVEFLHKYNFDGL
chitinase	111	125	FLHKYNFDGLDIDWE
chitinase	116	130	NFDGLDIDWEYPGAS
chitinase	121	135	DIDWEYPGASDRGGS
chitinase	126	140	YPGASDRGGSYGDRQ
chitinase	131	145	DRGGSYGDRQNFFYF
chitinase	136	150	YGDRQNFFYFVEELR
chitinase	141	155	NFFYFVEELRRAFDK
chitinase	146	160	VEELRRAFDKEGKGW
chitinase	151	165	RAFDKEGKGWEITMA
chitinase	156	170	EGKGWEITMAVPLAN
chitinase	161	175	EITMAVPLANFRLNE
chitinase	166	180	VPLANFRLNEGYHVP
chitinase	171	185	FRLNEGYHVPDLCEL
chitinase	176	190	GYHVPDLCELIDAVH
chitinase	181	195	DLCELIDAVHVMAYD
chitinase	186	200	IDAVHVMAYDLRGNW
chitinase	191	205	VMAYDLRGNWAGFAD
chitinase	196	210	LRGNWAGFADVHSPL
chitinase	201	215	AGFADVHSPLYQRPN

chitinase	206	220	VHSPLYQRPNEGYGY
chitinase	211	225	YQRPNEGYGYQALND
chitinase	216	230	EGYGYQALNDNDGMQ
chitinase	221	235	QALNDNDGMQLWVDK
chitinase	226	240	NDGMQLWVDKGCSPD
chitinase	231	245	LWVDKGCSPDKLVLG
chitinase	236	250	GCSPDKLVLGTPFYG
chitinase	241	255	KLVLGTPFYGRTFTL
chitinase	246	260	TPFYGRTFTLSQGNT
chitinase	251	265	RTFTLSQGNTNKDIG
chitinase	256	270	SQGNTNKDIGTYINK
chitinase	261	275	NKDIGTYINKDAGGG
chitinase	266	280	TYINKDAGGGDAGPY
chitinase	271	285	DAGGGDAGPYTGAKG
chitinase	276	290	DAGPYTGAKGMLAYY
chitinase	281	295	TGAKGMLAYYEICNM
chitinase	286	300	MLAYYEICNMLQVNA
chitinase	291	305	EICNMLQVNASKWTQ
chitinase	296	310	LQVNASKWTQKFDDI
chitinase	301	315	SKWTQKFDDIGKCPY
chitinase	306	320	KFDDIGKCPYAYDDG
chitinase	311	325	GKCPYAYDDGNQWVG
chitinase	316	330	AYDDGNQWVGYDNEI
chitinase	321	335	NQWVGYDNEISLQYK
chitinase	326	340	YDNEISLQYKMDFIK
chitinase	331	345	SLQYKMDFIKEKGYL
chitinase	336	350	MDFIKEKGYLGAMTW
chitinase	341	355	EKGYLGAMTWAIDMD
chitinase	346	360	GAMTWAIDMDDFHGT
chitinase	351	365	AIDMDDFHGTCGQKN
chitinase	356	370	DFHGTCGQKNPLINV
chitinase	361	375	CGQKNPLINVLAKNM
chitinase	366	380	PLINVLAKNMKDYVV
chitinase	371	385	LAKNMKDYVVPTLQI
chitinase	376	390	KDYVVPTLQISTTPR
chitinase	381	395	PTLQISTTPRPEWDR
chitinase	386	400	STTPRPEWDRPKSTT
chitinase	391	405	PEWDRPKSTTFEGGS
chitinase	396	410	PKSTTFEGGSVTTST
chitinase	401	415	FEGGSVTTSTTTTTT
chitinase	406	420	VTTSTTTTTTMKTTI
chitinase	411	425	TTTTTMKTTIPETTT
chitinase	416	430	MKTTIPETTTTGTTT

chitinase	421	435	PETTTTGTTTSTIDP
chitinase	426	440	TGTTTSTIDPTITTP
chitinase	431	445	STIDPTITTPSFPPS
chitinase	436	450	TITTPSFPPSETTTD
chitinase	441	455	SFPPSETTTDATTGG
chitinase	446	460	ETTTDATTGGPTVTP
chitinase	451	465	ATTGGPTVTPSCANA
chitinase	456	470	PTVTPSCANANFYPA
chitinase	461	475	SCANANFYPAANCNQ
chitinase	466	480	NFYPAANCNQYYMCN
chitinase	471	485	ANCNQYYMCNQGTPI
chitinase	476	490	YYMCNQGTPILMTCP
chitinase	481	495	QGTPILMTCPSGTVW
chitinase	486	500	LMTCPSGTVWVQEGI
chitinase	491	505	SGTVWVQEGIRCDWP
chitinase	496	510	VQEGIRCDWPAASTR
chitinase	501	515	RCDWPAASTRAECRS
chitinase	502	516	CDWPAASTRAECRSA
vitellogenin	1	15	ITPGWLPINSQLDYH
vitellogenin	6	20	LPINSQLDYHVHGRT
vitellogenin	11	25	QLDYHVHGRTFSSLF
vitellogenin	16	30	VHGRTFSSLFQVANQ
vitellogenin	21	35	FSSLFQVANQYTGIL
vitellogenin	26	40	QVANQYTGILYKARL
vitellogenin	31	45	YTGILYKARLSLDRN
vitellogenin	36	50	YKARLSLDRNEDQLI
vitellogenin	41	55	SLDRNEDQLITGKVT
vitellogenin	46	60	EDQLITGKVTEAQFS
vitellogenin	51	65	TGKVTEAQFSPVNTQ
vitellogenin	56	70	EAQFSPVNTQFSSGW
vitellogenin	61	75	PVNTQFSSGWDESVP
vitellogenin	66	80	FSSGWDESVPDEKLH
vitellogenin	71	85	DESVPDEKLHWDVVP
vitellogenin	76	90	DEKLHWDVVPMSQQP
vitellogenin	81	95	WDVVPMSQQPFQIEL
vitellogenin	86	100	MSQQPFQIELNSRGE
vitellogenin	91	105	FQIELNSRGEVRKLR
vitellogenin	96	110	NSRGEVRKLRVNKFV
vitellogenin	101	115	VRKLRVNKFVELWEI
vitellogenin	106	120	VNKFVELWEINMIKA
vitellogenin	111	125	ELWEINMIKAIISQL
vitellogenin	116	130	NMIKAIISQLQVVVD
vitellogenin	121	135	IISQLQVVVDEDKKV

vitellogenin	126	140	QVVVDEDKKVYRVFE
vitellogenin	131	145	EDKKVYRVFESTVTG
vitellogenin	136	150	YRVFESTVTGRCEAL
vitellogenin	141	155	STVTGRCEALYEVDH
vitellogenin	146	160	RCEALYEVDHLYPTT
vitellogenin	151	165	YEVDHLYPTTYLNPW
vitellogenin	156	170	LYPTTYLNPWQWTQQ
vitellogenin	161	175	YLNPWQWTQQHDTKL
vitellogenin	166	180	QWTQQHDTKLRIMKT
vitellogenin	171	185	HDTKLRIMKTHQFTN
vitellogenin	176	190	RIMKTHQFTNCRHNS
vitellogenin	181	195	HQFTNCRHNSAYKLH
vitellogenin	186	200	CRHNSAYKLHFNAFE
vitellogenin	191	205	AYKLHFNAFEYFHLK
vitellogenin	196	210	FNAFEYFHLKQHKPE
vitellogenin	201	215	YFHLKQHKPETFLSN
vitellogenin	206	220	QHKPETFLSNSAVSR
vitellogenin	211	225	TFLSNSAVSRVIADG
vitellogenin	216	230	SAVSRVIADGDNLKN
vitellogenin	221	235	VIADGDNLKNFTFYS
vitellogenin	226	240	DNLKNFTFYSGETIH
vitellogenin	231	245	FTFYSGETIHKIVLN
vitellogenin	236	250	GETIHKIVLNPEIYN
vitellogenin	241	255	KIVLNPEIYNKQKGM
vitellogenin	246	260	PEIYNKQKGMLVSHI
vitellogenin	251	265	KQKGMLVSHINVTVE
vitellogenin	256	270	LVSHINVTVERKGRE
vitellogenin	261	275	NVTVERKGRELTVID
vitellogenin	266	280	RKGRELTVIDYELRN
vitellogenin	271	285	LTVIDYELRNVGDLS
vitellogenin	276	290	YELRNVGDLSYSTSL
vitellogenin	281	295	VGDLSYSTSLVKAHS
vitellogenin	286	300	YSTSLVKAHSMRNSA
vitellogenin	291	305	VKAHSMRNSASMDLS
vitellogenin	296	310	MRNSASMDLSSSSMS
vitellogenin	301	315	SMDLSSSSMSSSSSS
vitellogenin	306	320	SSSMSSSSSSSSSSS
vitellogenin	311	325	SSSSSSSSSSSSSSS
vitellogenin	316	330	SSSSSSSSSSSSSSS
vitellogenin	321	335	SSSSSSSSSSSSEEH
vitellogenin	326	340	SSSSSSSEEHHSHNQ
vitellogenin	331	345	SSEEHHSHNQKLSKK
vitellogenin	336	350	HSHNQKLSKKRQVPL

vitellogenin	341	355	KLSKKRQVPLPRPLF
vitellogenin	346	360	RQVPLPRPLFEANFD
vitellogenin	351	365	PRPLFEANFDASSGL
vitellogenin	356	370	EANFDASSGLTTEQP
vitellogenin	361	375	ASSGLTTEQPVTFRP
vitellogenin	366	380	TTEQPVTFRPRRQLF
vitellogenin	371	385	VTFRPRRQLFQGQDM
vitellogenin	376	390	RRQLFQGQDMSEEET
vitellogenin	381	395	QGQDMSEEETEQNPE
vitellogenin	386	400	SEEETEQNPEIIPAN
vitellogenin	391	405	EQNPEIIPANLLPTY
vitellogenin	396	410	IIPANLLPTYNLIHN
vitellogenin	401	415	LLPTYNLIHNTKQVD
vitellogenin	406	420	NLIHNTKQVDVDPVG
vitellogenin	411	425	TKQVDVDPVGVAVRL
vitellogenin	416	430	VDPVGVAVRLSKDIA
vitellogenin	421	435	VAVRLSKDIAADLQG
vitellogenin	426	440	SKDIAADLQGEPRVG
vitellogenin	431	445	ADLQGEPRVGEDRHI
vitellogenin	436	450	EPRVGEDRHILPRFT
vitellogenin	441	455	EDRHILPRFTILVRL
vitellogenin	446	460	LPRFTILVRLLKQLK
vitellogenin	451	465	ILVRLLKQLKVSQIM
vitellogenin	456	470	LKQLKVSQIMEAARK
vitellogenin	461	475	VSQIMEAARKLYKLE
vitellogenin	466	480	EAARKLYKLENDHPN
vitellogenin	471	485	LYKLENDHPNYMNWD
vitellogenin	476	490	NDHPNYMNWDTWRVY
vitellogenin	481	495	YMNWDTWRVYRDAVS
vitellogenin	486	500	TWRVYRDAVSQAGTW
vitellogenin	491	505	RDAVSQAGTWSALNS
vitellogenin	496	510	QAGTWSALNSIQQFI
vitellogenin	501	515	SALNSIQQFISSEMV
vitellogenin	506	520	IQQFISSEMVEPKEA
vitellogenin	511	525	SSEMVEPKEASHLIT
vitellogenin	516	530	EPKEASHLITVLPAA
vitellogenin	521	535	SHLITVLPAAVSDKN
vitellogenin	526	540	VLPAAVSDKNKAYLH
vitellogenin	531	545	VSDKNKAYLHFLFEM
vitellogenin	536	550	KAYLHFLFEMTKDPV
vitellogenin	541	555	FLFEMTKDPVFKNMT
vitellogenin	546	560	TKDPVFKNMTYVNTS
vitellogenin	551	565	FKNMTYVNTSLVLAF

vitellogenin	556	570	YVNTSLVLAFSEVIH
vitellogenin	561	575	LVLAFSEVIHQVEMH
vitellogenin	566	580	SEVIHQVEMHQVRDL
vitellogenin	571	585	QVEMHQVRDLKIKSV
vitellogenin	576	590	QVRDLKIKSVYIPYL
vitellogenin	581	595	KIKSVYIPYLVQEFD
vitellogenin	586	600	YIPYLVQEFDDAVKE
vitellogenin	591	605	VQEFDDAVKENNSIK
vitellogenin	596	610	DAVKENNSIKIQLYT
vitellogenin	601	615	NNSIKIQLYTHALGV
vitellogenin	606	620	IQLYTHALGVTGNTH
vitellogenin	611	625	HALGVTGNTHILHYL
vitellogenin	616	630	TGNTHILHYLRPYII
vitellogenin	621	635	ILHYLRPYIIQLKTI
vitellogenin	626	640	RPYIIQLKTITHHQR
vitellogenin	631	645	QLKTITHHQRLFMVQ
vitellogenin	636	650	THHQRLFMVQSLERV
vitellogenin	641	655	LFMVQSLERVVEHNP
vitellogenin	646	660	SLERVVEHNPRKVID
vitellogenin	651	665	VEHNPRKVIDLLLSL
vitellogenin	656	670	RKVIDLLLSLYLDQN
vitellogenin	661	675	LLLSLYLDQNEHADI
vitellogenin	666	680	YLDQNEHADIRVEAL
vitellogenin	671	685	EHADIRVEALFLLMK
vitellogenin	676	690	RVEALFLLMKADPSI
vitellogenin	681	695	FLLMKADPSIHVLKM
vitellogenin	686	700	ADPSIHVLKMVAELT
vitellogenin	691	705	HVLKMVAELTHTESN
vitellogenin	696	710	VAELTHTESNNQVLS
vitellogenin	701	715	HTESNNQVLSASQSA
vitellogenin	706	720	NQVLSASQSAIKSAA
vitellogenin	711	725	ASQSAIKSAANVEGD
vitellogenin	716	730	IKSAANVEGDIYSEM
vitellogenin	721	735	NVEGDIYSEMRRKAK
vitellogenin	726	740	IYSEMRRKAKAVEHL
vitellogenin	731	745	RRKAKAVEHLLSTRN
vitellogenin	736	750	AVEHLLSTRNMDVSY
vitellogenin	741	755	LSTRNMDVSYSKSYL
vitellogenin	746	760	MDVSYSKSYLYGYKS
vitellogenin	751	765	SKSYLYGYKSKKINY
vitellogenin	756	770	YGYKSKKINYDSLYN
vitellogenin	761	775	KKINYDSLYNLNYIG
vitellogenin	766	780	DSLYNLNYIGSEDSI

vitellogenin	771	785	LNYIGSEDSIYPKSM
vitellogenin	776	790	SEDSIYPKSMLLNIF
vitellogenin	781	795	YPKSMLLNIFTNNLG
vitellogenin	786	800	LLNIFTNNLGRINTH
vitellogenin	791	805	TNNLGRINTHVQKGY
vitellogenin	796	810	RINTHVQKGYMVSSM
vitellogenin	801	815	VQKGYMVSSMTDLWE
vitellogenin	806	820	MVSSMTDLWEAFHTI
vitellogenin	811	825	TDLWEAFHTIYKKDN
vitellogenin	816	830	AFHTIYKKDNGSPTD
vitellogenin	821	835	YKKDNGSPTDPKTLV
vitellogenin	826	840	GSPTDPKTLVKFVEG
vitellogenin	831	845	PKTLVKFVEGNLKYF
vitellogenin	836	850	KFVEGNLKYFNMGVQ
vitellogenin	841	855	NLKYFNMGVQKFWAF
vitellogenin	846	860	NMGVQKFWAFDNTTF
vitellogenin	851	865	KFWAFDNTTFSNASA
vitellogenin	856	870	DNTTFSNASAVIQEF
vitellogenin	861	875	SNASAVIQEFLKTYK
vitellogenin	866	880	VIQEFLKTYKKPTNF
vitellogenin	871	885	LKTYKKPTNFNHTKL
vitellogenin	876	890	KPTNFNHTKLSSSSS
vitellogenin	881	895	NHTKLSSSSSITLTL
vitellogenin	886	900	SSSSSITLTLPCAMG
vitellogenin	891	905	ITLTLPCAMGLPAYF
vitellogenin	896	910	PCAMGLPAYFKMNSP
vitellogenin	901	915	LPAYFKMNSPSLWKY
vitellogenin	906	920	KMNSPSLWKYNGEFS
vitellogenin	911	925	SLWKYNGEFSIQTDA
vitellogenin	916	930	NGEFSIQTDAKTDVP
vitellogenin	921	935	IQTDAKTDVPMSLEN
vitellogenin	926	940	KTDVPMSLENFMNIT
vitellogenin	931	945	MSLENFMNITGSINL
vitellogenin	936	950	FMNITGSINLMFSQM
vitellogenin	941	955	GSINLMFSQMYHAQL
vitellogenin	946	960	MFSQMYHAQLAFSTA
vitellogenin	951	965	YHAQLAFSTAFDNKE
vitellogenin	956	970	AFSTAFDNKEYISGL
vitellogenin	961	975	FDNKEYISGLDRKVE
vitellogenin	966	980	YISGLDRKVEVHVPV
vitellogenin	971	985	DRKVEVHVPVKFQIN
vitellogenin	976	990	VHVPVKFQINLDFKN
vitellogenin	981	995	KFQINLDFKNHNGFI

vitellogenin	986	1000	LDFKNHNGFIRIIPL
vitellogenin	991	1005	HNGFIRIIPLFTDRD
vitellogenin	996	1010	RIIPLFTDRDYDVLQ
vitellogenin	1001	1015	FTDRDYDVLQWQTIP
vitellogenin	1006	1020	YDVLQWQTIPYTTIH
vitellogenin	1011	1025	WQTIPYTTIHNVPDF
vitellogenin	1016	1030	YTTIHNVPDFETVYM
vitellogenin	1021	1035	NVPDFETVYMDQLFK
vitellogenin	1026	1040	ETVYMDQLFKLIHVR
vitellogenin	1031	1045	DQLFKLIHVRKTAHF
vitellogenin	1036	1050	LIHVRKTAHFEKKMG
vitellogenin	1041	1055	KTAHFEKKMGENTGI
vitellogenin	1046	1060	EKKMGENTGIVFKVK
vitellogenin	1051	1065	ENTGIVFKVKYDTDQ
vitellogenin	1056	1070	VFKVKYDTDQEFLDT
vitellogenin	1061	1075	YDTDQEFLDTKWFLD
vitellogenin	1066	1080	EFLDTKWFLDEFKVL
vitellogenin	1071	1085	KWFLDEFKVLQLFTG
vitellogenin	1076	1090	EFKVLQLFTGLNYDV
vitellogenin	1081	1095	QLFTGLNYDVPTKDI
vitellogenin	1086	1100	LNYDVPTKDIFYNNL
vitellogenin	1091	1105	PTKDIFYNNLTVYYD
vitellogenin	1096	1110	FYNNLTVYYDHEDTK
vitellogenin	1101	1115	TVYYDHEDTKNHAVS
vitellogenin	1106	1120	HEDTKNHAVSFTVTK
vitellogenin	1111	1125	NHAVSFTVTKEQSKF
vitellogenin	1116	1130	FTVTKEQSKFYETLN
vitellogenin	1121	1135	EQSKFYETLNPVVQQ
vitellogenin	1126	1140	YETLNPVVQQNLKLS
vitellogenin	1131	1145	PVVQQNLKLSSGKKQ
vitellogenin	1136	1150	NLKLSSGKKQKHRNV
vitellogenin	1141	1155	SGKKQKHRNVKSHRI
vitellogenin	1146	1160	KHRNVKSHRIRREYT
vitellogenin	1151	1165	KSHRIRREYTEDENP
vitellogenin	1156	1170	RREYTEDENPAIPKD
vitellogenin	1161	1175	EDENPAIPKDKQPNS
vitellogenin	1166	1180	AIPKDKQPNSHPRRQ
vitellogenin	1171	1185	KQPNSHPRRQEYLSK
vitellogenin	1176	1190	HPRRQEYLSKSMALT
vitellogenin	1181	1195	EYLSKSMALTGDATA
vitellogenin	1186	1200	SMALTGDATAVVLDM
vitellogenin	1191	1205	GDATAVVLDMTLKFE
vitellogenin	1196	1210	VVLDMTLKFEGPAES

vitellogenin	1201	1215	TLKFEGPAESYFTTT
vitellogenin	1206	1220	GPAESYFTTTVSHAT
vitellogenin	1211	1225	YFTTTVSHATSLVNG
vitellogenin	1216	1230	VSHATSLVNGSSNYL
vitellogenin	1221	1235	SLVNGSSNYLLFYDQ
vitellogenin	1226	1240	SSNYLLFYDQHYYEE
vitellogenin	1231	1245	LFYDQHYYEEKKRNQ
vitellogenin	1236	1250	HYYEEKKRNQFCLSW
vitellogenin	1241	1255	KKRNQFCLSWSVYKP
vitellogenin	1246	1260	FCLSWSVYKPQVPIM
vitellogenin	1251	1265	SVYKPQVPIMNIYSA
vitellogenin	1256	1270	QVPIMNIYSAFEFDP
vitellogenin	1261	1275	NIYSAFEFDPNSKVH
vitellogenin	1266	1280	FEFDPNSKVHAIMNI
vitellogenin	1271	1285	NSKVHAIMNIGKECE
vitellogenin	1276	1290	AIMNIGKECENGGSA
vitellogenin	1281	1295	GKECENGGSAVANID
vitellogenin	1286	1300	NGGSAVANIDMLRLS
vitellogenin	1291	1305	VANIDMLRLSEHLDY
vitellogenin	1296	1310	MLRLSEHLDYVKNLT
vitellogenin	1301	1315	EHLDYVKNLTVSKLC
vitellogenin	1306	1320	VKNLTVSKLCDHEMR
vitellogenin	1311	1325	VSKLCDHEMRTKRDH
vitellogenin	1316	1330	DHEMRTKRDHVLPAC
vitellogenin	1321	1335	TKRDHVLPACRNSTE
vitellogenin	1326	1340	VLPACRNSTERASDL
vitellogenin	1331	1345	RNSTERASDLNRVHV
vitellogenin	1336	1350	RASDLNRVHVDINYN
vitellogenin	1341	1355	NRVHVDINYNLKQHE
vitellogenin	1346	1360	DINYNLKQHETFKRR
vitellogenin	1351	1365	LKQHETFKRRVYKVY
vitellogenin	1356	1370	TFKRRVYKVYDFVRT
vitellogenin	1361	1375	VYKVYDFVRTHLYPH
vitellogenin	1366	1380	DFVRTHLYPHVSEDV
vitellogenin	1371	1385	HLYPHVSEDVIVDNP
vitellogenin	1376	1390	VSEDVIVDNPAQFIS
vitellogenin	1381	1395	IVDNPAQFISANFTL
vitellogenin	1386	1400	AQFISANFTLKDNTR
vitellogenin	1391	1405	ANFTLKDNTRAFNVS
vitellogenin	1396	1410	KDNTRAFNVSIETPV
vitellogenin	1401	1415	AFNVSIETPVLSVNA
vitellogenin	1406	1420	IETPVLSVNATSVRL
vitellogenin	1411	1425	LSVNATSVRLQSWQS

vitellogenin	1416	1430	TSVRLQSWQSEMLRM
vitellogenin	1421	1435	QSWQSEMLRMNPRTS
vitellogenin	1426	1440	EMLRMNPRTSFAKRF
vitellogenin	1431	1445	NPRTSFAKRFAKWAL
vitellogenin	1436	1450	FAKRFAKWALPLYYK
vitellogenin	1441	1455	AKWALPLYYKPTCVV
vitellogenin	1446	1460	PLYYKPTCVVDSSYI
vitellogenin	1451	1465	PTCVVDSSYINTFDN
vitellogenin	1456	1470	DSSYINTFDNFTYSA
vitellogenin	1461	1475	NTFDNFTYSAHHIVQ
vitellogenin	1466	1480	FTYSAHHIVQNDAFY
vitellogenin	1471	1485	HHIVQNDAFYTILDI
vitellogenin	1476	1490	NDAFYTILDIPQKFN
vitellogenin	1481	1495	TILDIPQKFNMEYFK
vitellogenin	1486	1500	PQKFNMEYFKVAFKP
vitellogenin	1491	1505	MEYFKVAFKPTSPVP
vitellogenin	1496	1510	VAFKPTSPVPNMQRE
vitellogenin	1501	1515	TSPVPNMQREVLVFL
vitellogenin	1506	1520	NMQREVLVFLRNAKI
vitellogenin	1511	1525	VLVFLRNAKIELKPN
vitellogenin	1516	1530	RNAKIELKPNQGMPE
vitellogenin	1521	1535	ELKPNQGMPEVYVEG
vitellogenin	1526	1540	QGMPEVYVEGKRVDY
vitellogenin	1531	1545	VYVEGKRVDYNHHHS
vitellogenin	1536	1550	KRVDYNHHHSTDLNV
vitellogenin	1541	1555	NHHHSTDLNVSQDRI
vitellogenin	1546	1560	TDLNVSQDRIGYVYA
vitellogenin	1551	1565	SQDRIGYVYALPTKA
vitellogenin	1556	1570	GYVYALPTKAAHIVF
vitellogenin	1561	1575	LPTKAAHIVFPSYEI
vitellogenin	1566	1580	AHIVFPSYEIEMFYD
vitellogenin	1571	1585	PSYEIEMFYDGSRIM
vitellogenin	1576	1590	EMFYDGSRIMIQASN
vitellogenin	1581	1595	GSRIMIQASNMYRNF
vitellogenin	1586	1600	IQASNMYRNFTKGLC
vitellogenin	1591	1605	MYRNFTKGLCGNMDG
vitellogenin	1596	1610	TKGLCGNMDGEFVND
vitellogenin	1601	1615	GNMDGEFVNDVLTPW
vitellogenin	1606	1620	EFVNDVLTPWGCYAK
vitellogenin	1611	1625	VLTPWGCYAKDMALF
vitellogenin	1616	1630	GCYAKDMALFVASYA
vitellogenin	1621	1635	DMALFVASYADNSNS
vitellogenin	1626	1640	VASYADNSNSEVRKI

vitellogenin	1631	1645	DNSNSEVRKIKATQN
vitellogenin	1636	1650	EVRKIKATQNEQTCV
vitellogenin	1641	1655	KATQNEQTCVPQFHQ
vitellogenin	1646	1660	EQTCVPQFHQPLVSH
vitellogenin	1651	1665	PQFHQPLVSHQMRLS
vitellogenin	1656	1670	PLVSHQMRLSQVIKL
vitellogenin	1661	1675	QMRLSQVIKLADTSS
vitellogenin	1666	1680	QVIKLADTSSSSESS
vitellogenin	1671	1685	ADTSSSSESSSSSES
vitellogenin	1676	1690	SSESSSSSESHENNS
vitellogenin	1681	1695	SSSESHENNSSPSSE
vitellogenin	1686	1700	HENNSSPSSESQVNK
vitellogenin	1691	1705	SPSSESQVNKSKRQP
vitellogenin	1696	1710	SQVNKSKRQPNSRPR
vitellogenin	1701	1715	SKRQPNSRPRSSSSS
vitellogenin	1706	1720	NSRPRSSSSSSSSSS
vitellogenin	1711	1725	SSSSSSSSSSSESNE
vitellogenin	1716	1730	SSSSSSESNESVLAK
vitellogenin	1721	1735	SESNESVLAKKIINN
vitellogenin	1726	1740	SVLAKKIINNQIGPK
vitellogenin	1731	1745	KIINNQIGPKPTLIP
vitellogenin	1736	1750	QIGPKPTLIPSQSPM
vitellogenin	1741	1755	PTLIPSQSPMTSDDK
vitellogenin	1746	1760	SQSPMTSDDKCMTQQ
vitellogenin	1751	1765	TSDDKCMTQQPRHTY
vitellogenin	1756	1770	CMTQQPRHTYYENQF
vitellogenin	1761	1775	PRHTYYENQFCVSEK
vitellogenin	1766	1780	YENQFCVSEKPLDTC
vitellogenin	1771	1785	CVSEKPLDTCMPLIC
vitellogenin	1776	1790	PLDTCMPLICHATES
vitellogenin	1781	1795	MPLICHATESYTIDV
vitellogenin	1786	1800	HATESYTIDVNFYCV
vitellogenin	1791	1805	YTIDVNFYCVPLGPA
vitellogenin	1796	1810	NFYCVPLGPAANHYM
vitellogenin	1801	1815	PLGPAANHYMKLVKK
vitellogenin	1806	1820	ANHYMKLVKKGILPD
vitellogenin	1811	1825	KLVKKGILPDLSRNR
vitellogenin	1816	1830	GILPDLSRNRNGKRV
vitellogenin	1821	1835	LSRNRNGKRVVLPVE
vitellogenin	1826	1840	NGKRVVLPVEIPIQC
vitellogenin	1831	1845	VLPVEIPIQCEPVLN
