# Supplementary material for: IgE and T Cell Reactivity to a Comprehensive Panel of Cockroach Allergens in Relation to Disease
Source: Front Immunol. 2021 Feb 10;11:621700. doi: 10.3389/fimmu.2020.621700 (PMC7902920; doi:10.3389/fimmu.2020.621700)
Supplement: Supplementary file 5 [file Table_2.pdf]

Supplemental Table 2. Antibodies used for AIM assays

Target	Fluorochrome	Clone	Host	Company
CD4	APC-eFluor 780	RPA-T4	Mouse	Invitrogen
CD3	Alexa Fluor 700	UCHT1	Mouse	Invitrogen
CD8	V500	RPA-T8	Mouse	BD
CD14	V500	M5E2	Mouse	BD
CD19	V500	HIB19	Mouse	BD
CD25	APC	BC96	Mouse	Invitrogen
OX40 (CD134)	BV421	Ber-ACT35	Mouse	Biolegend
CD45RO	FITC	UCHL1	Mouse	Invitrogen
PDL-1 (CD274)	PE	29E2A3	Mouse	Biolegend
live/dead	Aqua (V500)-	-	-	Thermo Fisher
