# Supplementary material for: IgE and T Cell Reactivity to a Comprehensive Panel of Cockroach Allergens in Relation to Disease
Source: Front Immunol. 2021 Feb 10;11:621700. doi: 10.3389/fimmu.2020.621700 (PMC7902920; doi:10.3389/fimmu.2020.621700)
Supplement: Supplementary file 6 [file Table_3.pdf]

Supplemental Table 3. B and T-cell responses to cockroach allergens

Allergens:	Bla g 1	Bla g 2	Bla g 3	Bla g 4	Bla g 5	Bla g 6	Per a 7	Bla g 9	Bla g 11	Bla g 12	Vitellogenin	
**IgE antibody binding**	**Allergen-specific IgE (kU_A/L)**											**Sum**
Geometric mean	0.69	1.36	0.68	0.73	0.83	0.72	0.64	1.02	0.75	0.44	n.d.	12.07
**T cell responses**	**Activated cells per million CD4+ cells (fold)**											**Sum**
Geometric mean	1.55	1.64	1.80	1.26	1.02	1.37	1.22	2.01	2.01	1.38	5.06	24.73

n.d. not determined
